# Three New Resveratrol Derivatives from the Mangrove Endophytic Fungus *Alternaria* sp.

**DOI:** 10.3390/md12052840

**Published:** 2014-05-13

**Authors:** Jinhua Wang, Daniel G. Cox, Weijia Ding, Guanghao Huang, Yongcheng Lin, Chunyuan Li

**Affiliations:** 1 Institute of Biomaterial, College of Science, South China Agricultural University, Guangzhou 510642, China; E-Mails: alanwongjh@sina.com (J.W.); dwjzsu@scau.edu.cn (W.D.); ghhscau@sina.com (G.H.); chunyuanli@scau.edu.cn (C.L.); 2 Department of Pharmacognosy and Research Institute of Pharmaceutical Sciences, School of Pharmacy, Mississippi University, Oxford, MS 38677, USA; E-Mail: dgcox@go.olemiss.edu; 3 School of Chemistry and Chemical Engineering, Sun Yat-sen University, 135 Xingang West Road, Guangzhou 510275, China; E-Mail: ceslyc@mail.sysu.edu.cn

**Keywords:** marine fungus, *Alternaria* sp., mangrove, stilbenes, resveratrol, antioxidant, antitumor

## Abstract

Three new resveratrol derivatives, namely, resveratrodehydes A–C (**1**–**3**), were isolated from the mangrove endophytic fungus *Alternaria* sp. R6. The structures of these compounds were elucidated by analysis of their MS, 1D and 2D NMR spectroscopic data. All compounds showed broad-spectrum inhibitory activities against three human cancer cell lines including human breast MDA-MB-435, human liver HepG2, and human colon HCT-116 by MTT assay (IC_5__0_ < 50 μM). Among them, compounds **1** and **2** both exhibited marked cytotoxic activities against MDA-MB-435 and HCT-116 cell lines (IC_50_ < 10 μM). Additionally, compounds **1** and **3** showed moderate antioxidant activity by DPPH radical scavenging assay.

## 1. Introduction

Stilbenes are structurally characterized by the presence of 1,2-diphenylethylene nucleus and can be divided into two categories, the monomeric and oligomeric stilbenes. These compounds and their derivatives are of significant interest for drug research and development due to their therapeutic and preventive potential, exemplified by resveratrol and combretastatin A-4 [1]. Resveratrol (3,5,4′-trihydroxystilbene) is a widely recognized compound that is known to prevent or slow the development of some diseases *in vitro/vivo* studies, including cancer [2], cardiovascular disease [3] and ischemic injuries [4]. There are also some preclinical evidences that resveratrol can enhance stress resistance [5] and extend the lifespan of various organisms ranging from yeasts to vertebrates [6]. Resveratrol has been found in grapes (*Vitis vinifera*), a variety of other berries, peanuts, and medicinal plants such as Japanese knotweed (*Polygonum cuspidatum* Siebold and Zucc.) [7] and several genera of fungi including, *Botryosphaeria*, *Penicillium*, *Cephalosporium*, *Aspergillus*, *Geotrichum*, *Mucor*, and *Alternaria* [8].

**Figure 1:**
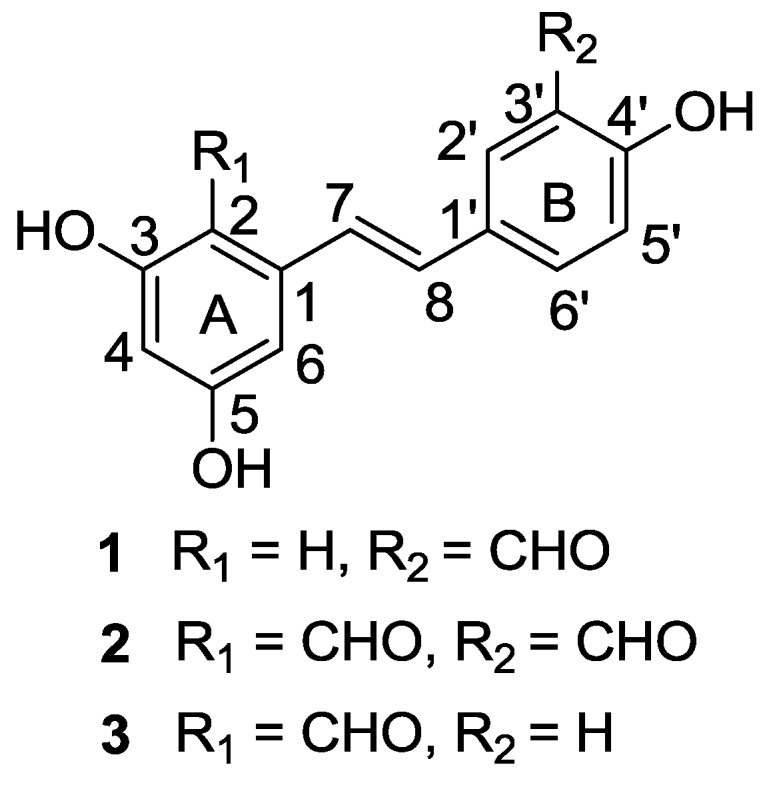
Structures of compounds **1**–**3**.

*Alternaria* is a cosmopolitan fungal genus, and several species are known as plant pathogens. Although several endophytic *Alternaria* strains have been investigated [9,10], isolates from particular habitats frequently yield novel natural products. This prompted us to investigate the metabolites produced by the mangrove-derived endophytic *Alternaria* sp. Three new resveratrol derivatives, namely, resveratrodehydes A–C (**1**–**3**) (Figure 1), were isolated and elucidated from the culture of the fungus *Alternaria* sp. (collection No. R6), which was obtained from the root of a mangrove plant *Myoporum bontioides* A. Gray. Details of the isolation, structure elucidation, and biological activity of compounds **1**–**3** are reported herein.

## 2. Results and Discussion

### 2.1. Chemical Structure Elucidation

Compound **1** was obtained as a yellow needle, with a high-resolution deprotonated molecular ion peak at *m*/*z* 255.0664 [M − H]^+^, consistent with a molecular formula of C_15_H_12_O_4_. The ^1^H NMR spectrum of **1** (Table 1) displayed signals suggestive of a 1′,3′,4′-trisubstituted aromatic ring at *δ*_H_ 7.95 (d, 2.0 Hz, 1H), 7.84 (dd, 8.4, 2.0 Hz, 1H), and 7.00 (d, 8.4 Hz, 1H), a *trans*-double bond at *δ*_H_ 7.11 (d, 16.2 Hz, 1H) and 7.04 (d, 16.2 Hz, 1H), and a 1, 3, 5-trisubstituted aromatic ring at *δ*_H_ 6.57 (d, 2.4 Hz, 2H) and 6.31 (t, 2.4 Hz, 1H). Additionally, the ^1^H NMR spectrum of **1** also revealed the signals of one aldehyde substituent at *δ*_H_ 10.07 (s, 1H), and three hydroxyl groups at *δ*_H_ 8.36 (s, 2H, exchangable) and 10.96 (s, 1H, exchangable), respectively. The signal of the aldehyde group was also apparent in the ^13^C NMR spectrum (Table 1) at *δ*c 197.0 confirmed by the HSQC correlation. The remaining ^13^C NMR signals could be assigned to two aromatic rings and one trans-double bond by analysis of the ^13^C NMR and HSQC spectra of **1**. This suggested that compound **1** is an aldehydated stilbene derivative. In the ^1^H NMR spectrum of **1**, H-2 and H-6 appeared at *δ*_H_ 6.57 (d, 2.4Hz, 2H). Furthermore, the ^13^C NMR signals for C-2 and C-6, and C-3 and C-5, of compound **1** were evident at *δ*_C_ 105.0 and 158.8, respectively indicated by the HMBC correlations from the protons at *δ*_H_ 6.57 to the carbons at *δ*_C _158.8, 102.3 and 128.2 (C-7), and from the hydroxyls at *δ*_H_ 8.36 (s, 2H, exchangable) to the carbons at *δ*_C_ 158.8, 102.3 and 105.0. Therefore, two hydroxyl groups attached to C-3 and C-5 could be deduced. These data, in combination with the observed key HMBC correlations of the proton of 3′-CHO to C-2′, C-3′, and C-4′, H-7 to C-1′, C-2, and C-6, and H-8 to C-1, C-2′, and C-6′, permitted the determination of the structure of **1** as 3,5,4′-trihydroxy-3′-formyl-trans-stilbene.

The molecular formula C_16_H_12_O_5_ was determined from the HRESIMS ([M − H]^+^ at *m*/*z* 283.0612, calcd for C_16_H_11_O_5_283.0612) for compound **2**. The ^1^H and ^13^C NMR spectral data (Table 1) of **2** were closely comparable to those of **1** and suggested the presence of a 1′,3′,4′-trisubstituted aromatic ring (ring B), a trans-double bond, and an aldehyde substituent in ring B of **2**, in a manner similar to **1**. However, the ^1^H NMR spectrum of **2** displayed only two doublets for aromatic ring A at *δ*_H_ 6.30 (d, 2.1 Hz, 1H) and 6.73 (d, 2.1 Hz, 1H), while three protons were present for this functionality (1, 3, 5-trisubstituted aromatic ring) in **1**. On the other hand, the ^13^C NMR spectra of 2 indicated there were two aldehyde (*δ*_C_ 196.8 and 193.4), and three oxygenated aromatic quaternary carbons (*δ*_C_ 166.1, 165.2 and 161.3) in 2, while only one aldehyde and three oxygenated aromatic quaternary carbons were evident in **1**. Furthermore, the molecular formula of **2** included an additional oxygen and carbon atom relative to that of **1**. All of above evidence suggested that compound **2** contains one more aldehyde group in the aromatic ring A compared to compound **1**. The locations of the aldehyde and hydroxy groups were determined on the basis of the observed HMBC correlations of the proton of 3′-CHO to C-2′, C-3′, and C-4′, H-5′ to C-1′, C-3′ and C-4′, H-8 to C-1′, C-2′, and C-6′, the proton of 2-CHO to C-2 and C-3, H-7 to C-1, C-2, and C-6, 3-OH to C-2, C-3 and C-4, and H-6 to C-2, C-4 and C-5. Thus, the structure of **2** was established as 3,5,4′-trihydroxy-2, 3′-diformyl-trans-stilbene.

Compound **3** shares the same molecular formula of C_15_H_12_O_4_ with **1**, as determined by the HRESIMS results ([M − H]^+^ at *m*/*z* 255.0559, calcd [M − H]^+^ 255.0663). The ^1^H and ^13^C NMR spectral data (Table 1) revealed **3** had the same trans-stilbene unit and substituents as **1**. Comprehensive comparison of the NMR spectra of **3** with that of **2** revealed their great similarity in ring A, which suggested an aldehyde group and two hydroxyls should be attached to C-2, C-3 and C-5 in the 1,2,3,5-tetrasubstituted benzene ring A, respectively. Additionally, the ^1^H NMR spectrum of **3** exhibited the signals of a set of ortho-coupled protons at *δ*_H_ 7.55 (d, 8.4 Hz, 2H) and 6.88 (d, 8.4 Hz, 2H), corresponding to the *p*-disubstituted phenyl ring B. Therefore, the remaining one hydroxyl was connected to C-4′ in B ring. The positions of the aldehyde and hydroxy groups were further confirmed according to the observed HMBC correlations of H-8 to C-1′, C-2′ and C-6′, H-7 to C-1, C-2, C-6, and C-1′, H-2′ to C-7, C-1′ and C-4′, the proton of 2-CHO to C-2 and C-3, 3-OH to C-2, C-3 and C-4, and H-6 to C-2, C-4 , C-5 and C-7.

The structures and key HMBC correlations of compounds **1**–**3** are shown in Figure 1 and Figure 2, respectively. More detailed spectra are available at the Supplementary Information (Figures S1–S24).

**Table 1:** ^1^H and ^13^C NMR data of compounds **1**–**3**, *J* in Hz.

Position	1 ^a^		2 ^a^		3 ^a^	
	*δ*_C_, mult.	*δ*_H_ (*J* in Hz)	*δ*_C_, mult.	*δ*_H_ (*J* in Hz)	*δ*_C_, mult.	*δ*_H_ (*J* in Hz)
1	139.3, C	-	144.9, C	-	145.6,C	-
2	105.0, CH	6.57 (d, 2.4)	112.0, C	-	112.0,C	-
3	158.8, C		166.1, C	-	166.2,C	-
4	102.3, CH	6.31 (t, 2.4)	101.8, CH	6.30 (d, 2.0)	101.4,CH	6.27 (d, 1.8)
5	158.8, C	-	165.2, C	-	165.2,C	-
6	105.0, C	6.57 (d, 2.4)	106.6, CH	6.73 (d, 2.0)	106.3,CH	6.70 (d, 1.8)
2-CHO	-	-	193.4, CH	10.33(s)	193.4,CH	10.33(s)
3-OH	-	8.36(s)	-	12.55(s)	-	12.58(s)
5-OH	-	8.36(s)	-	11.02(s)	-	9.74(s)
7	128.2, CH	7.04 (d, 16.2)	122.3, CH	7.83 (d, 16.2)	119.8, CH	7.67 (d, 16.2)
8	126.4, CH	7.11 (d, 16.2)	133.0, CH	7.17 (d, 16.2)	134.8, CH	7.08 (d, 15.6)
1′	129.9, C	-	129.3, C	-	128.6, C	-
2′	131.4, CH	7.95(d, 2.0)	132.4, CH	8.08 (d, 1.8)	128.6, CH	7.55 (d, 8.4)
3′	121.1, CH	-	121.1, C	-	115.6, CH	6.88 (d, 8.4)
4′	160.7, C	-	161.3, C	-	158.0, C	-
5′	117.5, CH	7.00 (d, 8.4)	117.6, CH	7.03 (d, 8.4)	115.6, CH	6.88 (d, 8.4)
6′	134.6, CH	7.84 (dd, 2.0, 8.4)	135.1, CH	7.97 (dd, 2.1, 8.4)	158.0, C	7.55 (d,8.4)
3′-CHO	197.0, CH	10.07(s)	196.8, CH	10.07(s)	-	-
4′-OH	-	10.96(s)	-	9.81(s)	-	8.69(s)

^a^ Measured in CD_3_COCD_3_ at 600 MHz (^1^H) and 150 MHz (^13^C).

**Figure 2:**
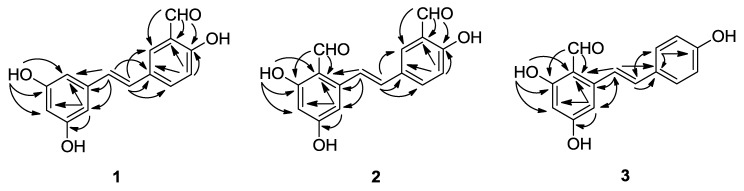
Key HMBC correlations of compounds **1**–**3**.

### 2.2. Biological Activity

**Figure 3:**
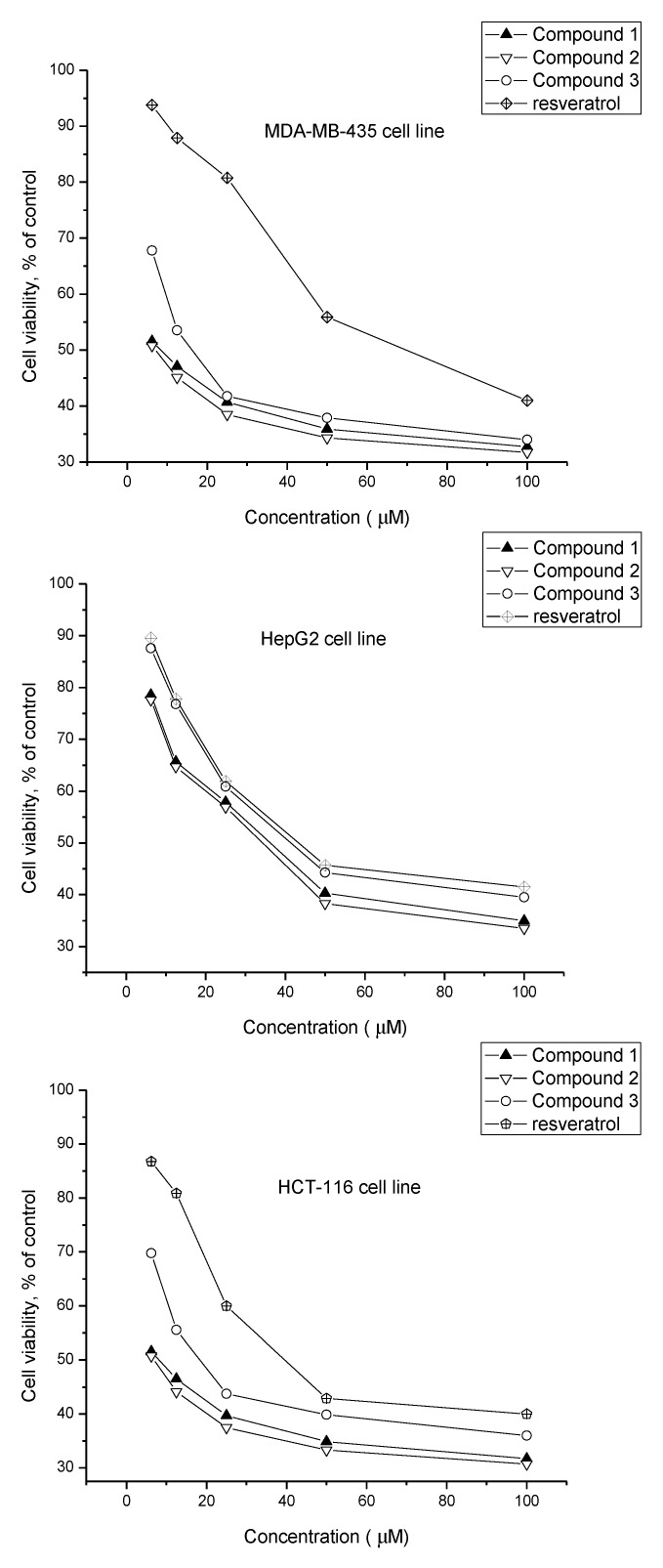
Effect of compounds **1**–**3** and resveratrol on cell lines proliferation (MDA-MB-435, HepG2, and HCT-116).

All of the three resveratrol analogues were tested on inhibitory activities against the growth of three carcinoma cell lines including estrogen-resistant human breast MDA-MB-435, human liver HepG2 and human colon HCT-116. As shown in Figure 3, continuous exposure to various concentrations of compounds **1**–**3** all resulted in dose-dependent decreases in the tested cell viability relative to the negative control cultures. From the Table 2, all of them showed broad-spectrum cytotoxic activities against tested cell lines. Moreover, the resveratrodehyde A (**1**) and resveratrodehyde B (**2**) exhibited marked cytotoxic activities against MDA-MB-435 and HCT-116 with IC_50_ values below 10 μM. In addition, all compounds displayed excellent selective activities against MDA-MB-435 and HCT-116 cell lines over the other cancer cell line. Although additional data will be required, the analysis of the *in vitro* cytotoxicity of resveratrodehydes A–C (**1**–**3**) should prove helpful in the study of their structure-activity relationships. Compound **1**–**3** all exhibited higher cytotoxic activities than resveratrol, which suggested that the -CHO substituents probably have effect in cytotoxic activities. Furthermore, compound **1** bears a -CHO group at C-3′ and exhibits greater cytotoxic activity than **3** which bears a -CHO group at C-2. Compound **2**, bearing a -CHO group at both C-2 and C-3′, exhibited cytotoxic activity similar to **1**, suggesting that the -CHO group at C-3′ probably was the key pharmacophore of reveratrodehyde for cytotoxic activities against tested cell lines.

As for the DPPH radical scavenging activity shown in Table 3, the IC_50_ values of compound **1**–**3** were 447.62 ± 5.00, >900.00 and 572.68 ± 6.41 μM, respectively, which were comparatively higher than the IC_50_ values of resveratrol (70.22 ± 0.35 μM). According to the data, compounds **1** and **3** share similar moderate DPPH scavenging activity which is lower than resveratrol and compound **2** showed only marginal activity. This result was in agreement with the previous reports that electron-withdrawing substituents, such as COOR and COOH, whether at the *para* or *ortho* position, stabilize the phenol form of antioxidants, and destabilize the phenoxy radical form of the antioxidants, to increase the O–H bond strength and make the antioxidants less efficient [11,12,13].

Stibenes are usually known as typical phytoalexins of some plants such as *Vitis vinifera* and *Picea sitchensis* involved in the defense of these plant species against fungal attack [14]. They also have been found in some genera of endophytic fungi including the *Alternaria* sp. [8]. The genus *Alternaria* is normally reported as a pathogen for many plants causing rotting or toxin production [15,16,17]. So, in this study, the new stilbenes producing by the endophytic *Alternaria* sp. (collection No. R6) maybe play the role of inhibiting other endophytes or invasive fungal in the mangrove plant *Myoporum bontioides*, which makes the *Alternaria* get more nutrition and living space. Further investigations are needed to fully reveal and confirm the exact role(s) of these stilbenes in *Alternaria* and in the *Alternaria*—mangrove interactions.

**Table 2:** Cytotoxicity (IC_50_, μM) of compounds **1**–**3** against MDA-MB-435, HepG2, and HCT-116 cell lines ^a^.

Samples	Cell lines		
	MDA-MB-435 ^c^	HepG2 ^c^	HCT-116 ^c^
**1**	8.56 ± 0.81	35.32 ± 1.67	7.82 ± 1.02
**2**	7.68 ± 0.90	32.70 ± 1.50	6.93 ± 0.63
**3**	16.49 ± 0.78	41.86 ± 0.57	18.63 ± 0.95
Resveratrol ^b^	68.56 ± 1.36	43.52 ± 1.29	38.87 ± 1.58
Epirubicin ^b^	0.56 ± 0.06	0.96 ± 0.02	0.48 ± 0.03

^a^ IC_50_ values are taken as means ± standard deviation from three independent experiment; ^b^ Used as a positive control. MDA-MB-435 ^c^ human breast cancer cell line; HepG2 ^c^ human liver cancer cell line; HCT-116 ^c^, human colon cancer cell line.

**Table 3:** DPPH radical scavenging activity of compounds **1–3** (IC_50_, μM) ^a^.

1	2	3	Resveratrol ^b^	Ascorbic acid ^b^
447.62 ± 5.00	>900.00	572.68 ± 6.41	70.22 ± 0.35	21.61 ± 0.00

^a^ IC_50_ values are taken as means ± standard deviation from three independent experiment; ^b^ Used as a positive control.

## 3. Experimental Section

### 3.1. General Experimental Procedures

Melting points were detected on an X-4 micromelting point apparatus (Cany Precision Instruments Co., Ltd., Shanghai, China), uncorrected. IR spectra were obtained on a Nicolet 5DX-Fourier transform infrared spectrophotometer (Thermo Electron Corporation, Madison, USA). All NMR experiments were recorded on a Bruker AVIII 600MHz NMR spectrometer (Bruker BioSpin GmbH company, Rheinstetten, Germany), using deuterated acetone as solvent and the residual solvent resonance as internal standard, and coupling constants (*J*) are in Hz. HR-ESI-MS were performed on LCMS-IT-TOF (Shimadzu, Janpan) mass spectrometer. Chromatography was carried out on a silica gel column (200–300 mesh; Qingdao haiyang chemicals Co., Ltd., Qingdao, China). All other reagents used were analytical grade.

### 3.2. Fungal Material and Fermentation

The strain of *Alternaria* sp. (collection No. R6) was isolated from the root of a marine semi-mangrove plant *Myoporum bontioides* A. Gray collected from the mangrove in Leizhou peninsula, Guangdong Province, China. Its species was identified by compared with the morphological characteristics with that of *Alternaria* sp. [8]. This fungus is stored in College of Science, South China Agricultural University, Guangzhou, China. Small agar slices bearing mycelia, were excised from stock cultures incubated at 25 °C on PDA (Potato Dextrose Agar) medium, and placed in GYT medium (1% glucose, 0.1% yeast extract, 0.2% peptone, 0.2% crude sea salt) in 500 mL Erlenmeyer flasks containing 250 mL GYT and incubated at 28 °C, 120 rpm for 6 days as seed culture. A 150 L large scale fermentation was performed using multiple 1000 mL Erlenmeyer flasks, each containing 500 mL GYT. Each flask was inoculated with 1 mL seed culture and incubated at 28 °C for 30 days under static conditions.

### 3.3. Extraction and Isolation

The resultant culture (150 L) was filtered through cheesecloth and the filtrate was concentrated to 5 L *in vacuo* below 50 °C and extracted five times by shaking with an equal volume of ethyl acetate. The combined extract (56.6 g) was subjected to silica gel column chromatography and eluted with petroleum ether–ethyl acetate (90:10, 80:20, 70:30, 50:50, and 0:100) to afford Fractions 1–5 (1.9, 3.6, 6.2, 8.7, and 15.8 g, respectively). Fraction 4 (8.7 g) was subjected to silica gel column chromatography and eluted with petroleum ether–ethyl acetate in a gradient of ethyl acetate (petroleum ether-ethyl acetate, 100:0–50:50). One hundred and twenty-two fractions of 20 mL each were collected, combined on the basis of their thin layer chromatography profiles, and concentrated to dryness to give thirteen major fractions A–M. Repeated slow recrystallization of fraction F (10 mg, petroleum ether-ethyl acetate 75:25, *v*/*v*), H(25 mg, petroleum ether-ethyl acetate 70:30, *v*/*v*), and I (10 mg, petroleum ether-ethyl acetate 65:35, *v*/*v*) at room temperature from petroleum ether-ethyl acetate (80:20, *v*/*v*) yielded 3.2 , 8.5, and 5.0 mg yellow crystalline needle shape solids of reveratrodehydes A–C (**1**–**3**), respectively.

Resveratrodehyde A (**1**): Yellow powder; mp: 178 °C–180 °C; IR (KBr) *ν*_max_ 3393, 3209, 2925, 2852, 1631.17, 1641, 1594, 1486, 1288, 1163, 952, 883 cm^−1^; ^1^H and ^13^C NMR data, see Table 1; HRESIMS *m*/*z* 255.0659 [M − H]^+^ (calcd. for C_1__5_H_12_O_4_, 255.0663).

Resveratrodehyde B (**2**): Yellow powder; mp: 238 °C–240 °C; IR (KBr) *ν*_max_ 3204, 2849, 2735, 1659, 1635, 1487, 1374, 1277, 1199, 1166, 956 cm^−1^; ^1^H and ^13^C NMR data, see Table 1; HRESIMS *m*/*z* 283.0612 [M − H]^+^ (calcd. for C_16_H_12_O_5_, 283.0612).

Resveratrodehyde C (**3**): Yellow powder; mp: 222 °C–224 °C; IR (KBr) *ν*_max_ 3287, 2822, 2707, 1635, 1595, 1303, 1248, 1157, 937, 812 cm^−1^; ^1^H and ^13^C NMR data, see Table 2; HRESIMS *m*/*z* 255.0664 [M − H]^+^ (calcd. for C_15_H_12_O_4_, 255.0663).

### 3.4. Cytotoxicity Assay

All compounds were evaluated for their *in vitro* cytotoxic activity against three human carcinoma cell lines including human colon HCT-116, human breast MDA-MB-435, and human liver HepG2 by MTT assay [18] using epirubicin (an anticancer drug used widely in the clinic [19,20,21], the IC_50_ value of epirubicin was from the literature [18]) and resveratrol as positive controls. The tested human cancer cell lines were harvested during logarithmic growth phase and seeded in 96-well plates at a density of 1 × 10^4^ cells/mL, and cultured at 37 °C in a humidified incubator (5% CO_2_) for 24 h, followed by exposure to various concentrations of each compound tested for 48 h. Negative control cells were incubated without test samples. Cells treated with epirubicin served as a positive control. Subsequently, 20 μL of MTT (3-(4,5-dimethylthiazol-2-yl)-2,5-diphenyl-2*H*-tetrazolium bromide) solution (5 mg/mL) was added to each well and mixed, the cells were then incubated for an additional 4 h. Culture supernatant was removed and 150 μL of dimethyl sulfoxide was added to each well to fully dissolve the MTT-formazan crystals. Cell growth inhibition was determined by measuring the absorbance (Abs) at λ = 570 nm using a microplate reader and calculated according to the following equation: Growth inhibition = (1 − OD of treated cells/OD of control cells) × 100%. The half maximal inhibitory concentrations (IC_50_) were obtained from liner regression analysis of the concentration-response curves plotted for each tested compound.

### 3.5. The DPPH Radical Scavenging Activity Assay

The DPPH radical scavenging activity was determined as the method described in previous literatures [22,23]. Briefly, 2 mL of ethanolic solution of the test compound was mixed with 2 mL of 0.16 mM ethanolic solution of DPPH. The mixture was shaken vigorously and incubated for 30 min at room temperature in the dark. The absorbance was measured at 517 nm. Ascorbic acid was taken as a positive control. The scavenging ability was calculated as: DPPH radical scavenging activity (%) = [1 − (A1 − A2)/A0] × 100, where A_0_ is the absorbance in the lack of the test compound, A_1_ is the absorbance in the presence of the test compound and DPPH, and A_2_ is the absorbance in the lack of DPPH. The IC_50_ values (the concentration of the antioxidant required to scavenge 50% of DPPH present in the test solution) were obtained from liner regression analysis of the concentration-response curves plotted for each tested compound.

## 4. Conclusions

This investigation led to the isolation of three new resveratrol derivatives, which enriched this family of compounds. This was the first report showing that stibene derivatives were produced by the endophytes of *Myoporum bontioides*. All the new stibenes have superior *in vitro* anti-tumor effects over resveratrol in MDA-MB-435, HCT-116 and HepG2 probably due to the substituent of aldehyde group. The marked selective cytotoxic activities of resveratrodehyde A (**1**) and resveratrodehyde B (**2**) against MDA-MB-435 and HCT-116 cell lines (IC_50_ < 10 μM) reveal that these resveratrol analogues are potential antitumor drug and/or lead compounds for constructing an antitumor compound library. On the contrary, the DPPH radical scavenging activity of resveratrodehyde A-C (IC_50_ > 400 μM) are much lower than that of resveratrol (IC_50_ = 70.22 μM), suggesting that these stibenes could have no importance as leads in studying on preventing or slowing the progress of aging and age-associated oxidative stress-related degenerative diseases.

## Supplementary Files

Supplementary File 1 Supplementary Information (PDF, 1642 KB)
