# Supplementary material for: Three New Resveratrol Derivatives from the Mangrove Endophytic Fungus Alternaria sp
Source: Mar Drugs. 2014 May 13;12(5):2840–50. doi: 10.3390/md12052840 (PMC4052320; doi:10.3390/md12052840)
Supplement: Supplementary File 1 — Supplementary Information (PDF, 1642 KB) [file marinedrugs-12-02840-s001.pdf]

Supplementary Information

Figure S1. ^{1}H NMR spectrum (600 MHz) of compound **1** in $(CD_3)_2CO$.
Figure S2. Expansion of ^{1}H NMR spectrum (600 MHz) of compound **1** in $(CD_3)_2CO$.
Figure S3. ^{13}C NMR spectrum (150 MHz) of compound **1** in $(CD_3)_2CO$.
Figure S4. HSQC spectrum (600/150 MHz) of compound **1** in $(CD_3)_2CO$.
Figure S5. HMBC spectrum (600/150 MHz) of compound **1** in $(CD_3)_2CO$.
Figure S6. Expansion of HMBC spectrum(600/150 MHz) of compound **1** in $(CD_3)_2CO$.
Figure S7. HRESIMS spectrum of compound **1**.
Figure S8. IR spectrum of compound **1**.
Figure S9. ^{1}H NMR spectrum (600 MHz) of compound **2** in $(CD_3)_2CO$.
Figure S10. Expansion of ^{1}H NMR spectrum (600 MHz) of compound **2** in $(CD_3)_2CO$.
Figure S11. ^{13}C NMR spectrum (150 MHz) of compound **2** in $(CD_3)_2CO$.
Figure S12. HSQC spectrum (600/150 MHz) of compound **2** in $(CD_3)_2CO$.
Figure S13. HMBC spectrum (600/150 MHz) of compound **2** in $(CD_3)_2CO$.
Figure S14. Expansion of HMBC spectrum(600/150 MHz) of compound **2** in $(CD_3)_2CO$.
Figure S15. HRESIMS spectrum of compound **2**.
Figure S16. IR spectrum of compound **2**.
Figure S17. ^{1}H NMR spectrum (600 MHz) of compound **3** in $(CD_3)_2CO$.
Figure S18. Expansion of ^{1}H NMR spectrum (600 MHz) of compound **3** in $(CD_3)_2CO$.
Figure S19. ^{13}C NMR spectrum (150 MHz) of compound **3** in $(CD_3)_2CO$.
Figure S20. HSQC spectrum (600/150 MHz) of compound **3** in $(CD_3)_2CO$.
Figure S21. HMBC spectrum (600/150 MHz) of compound **3** in $(CD_3)_2CO$.
Figure S22. Expansion of HMBC spectrum(600/150 MHz) of compound **3** in $(CD_3)_2CO$.
Figure S23. HRESIMS spectrum of compound **3**.
Figure S24. IR spectrum of compound **3**.

Figure S1. ^{1}H NMR spectrum (600 MHz) of compound **1** in $(CD_3)_2CO$.

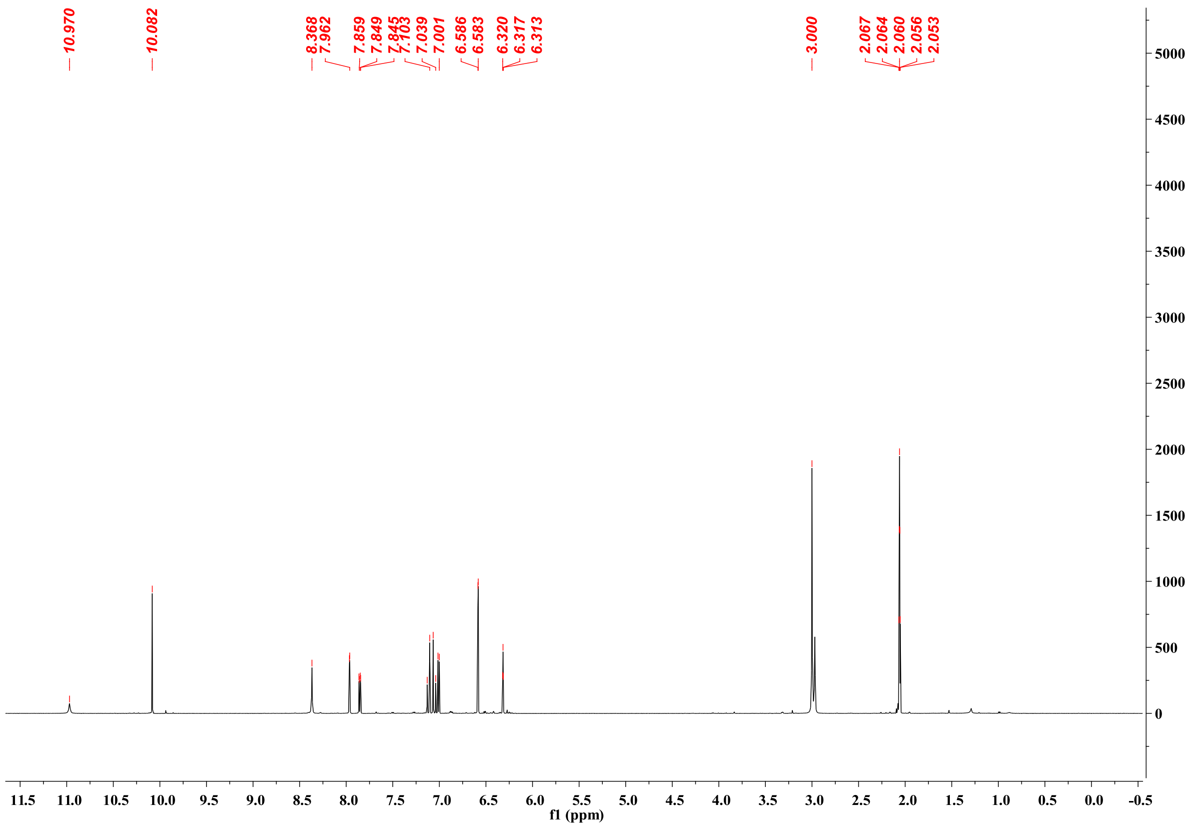

Figure S2. Expansion of ^{1}H NMR spectrum (600 MHz) of compound **1** in $(CD_3)_2CO$.

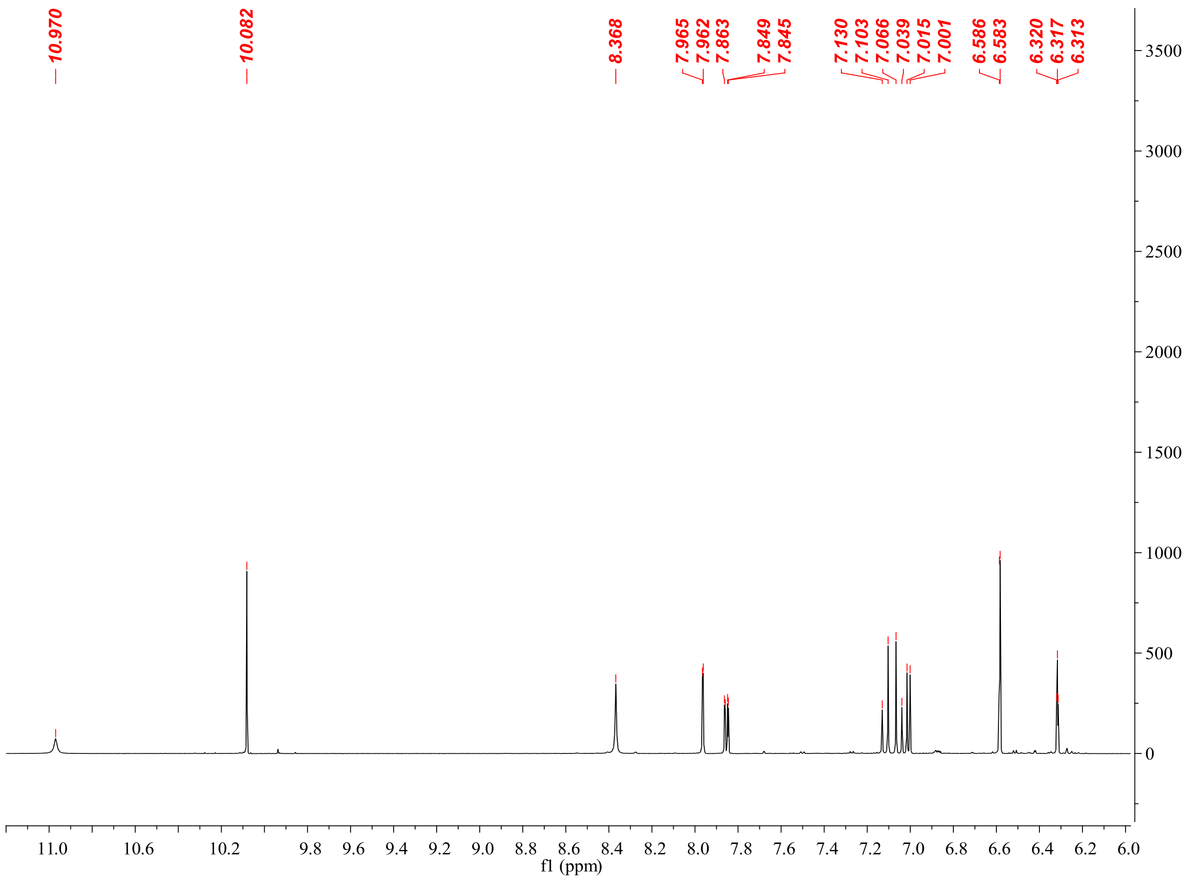

Figure S3. ^{13}C NMR spectrum (150 MHz) of compound **1** in $(CD_3)_2CO$.

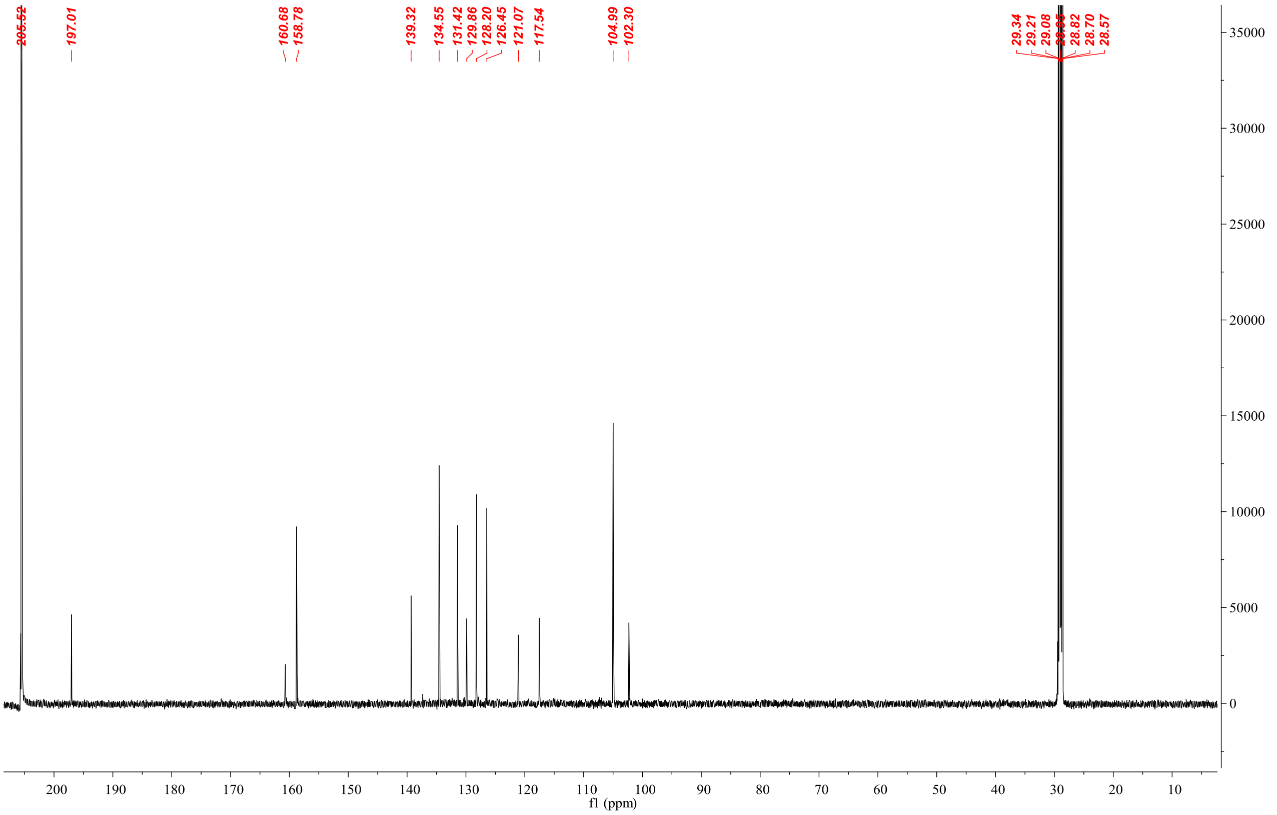

Figure S4. HSQC spectrum (600/150 MHz) of compound **1** in $(CD_3)_2CO$.

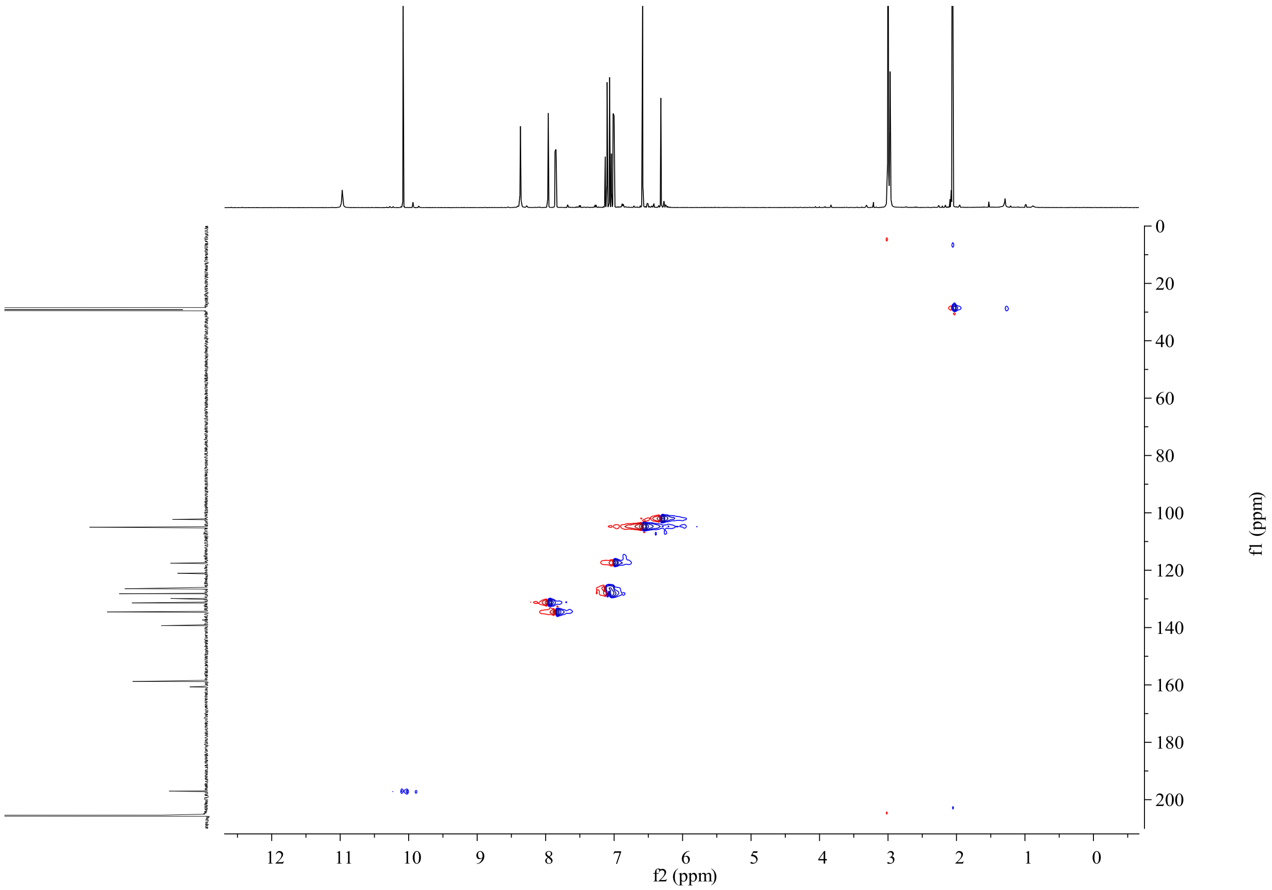

Figure S5. HMBC spectrum (600/150 MHz) of compound **1** in $(CD_3)_2CO$.

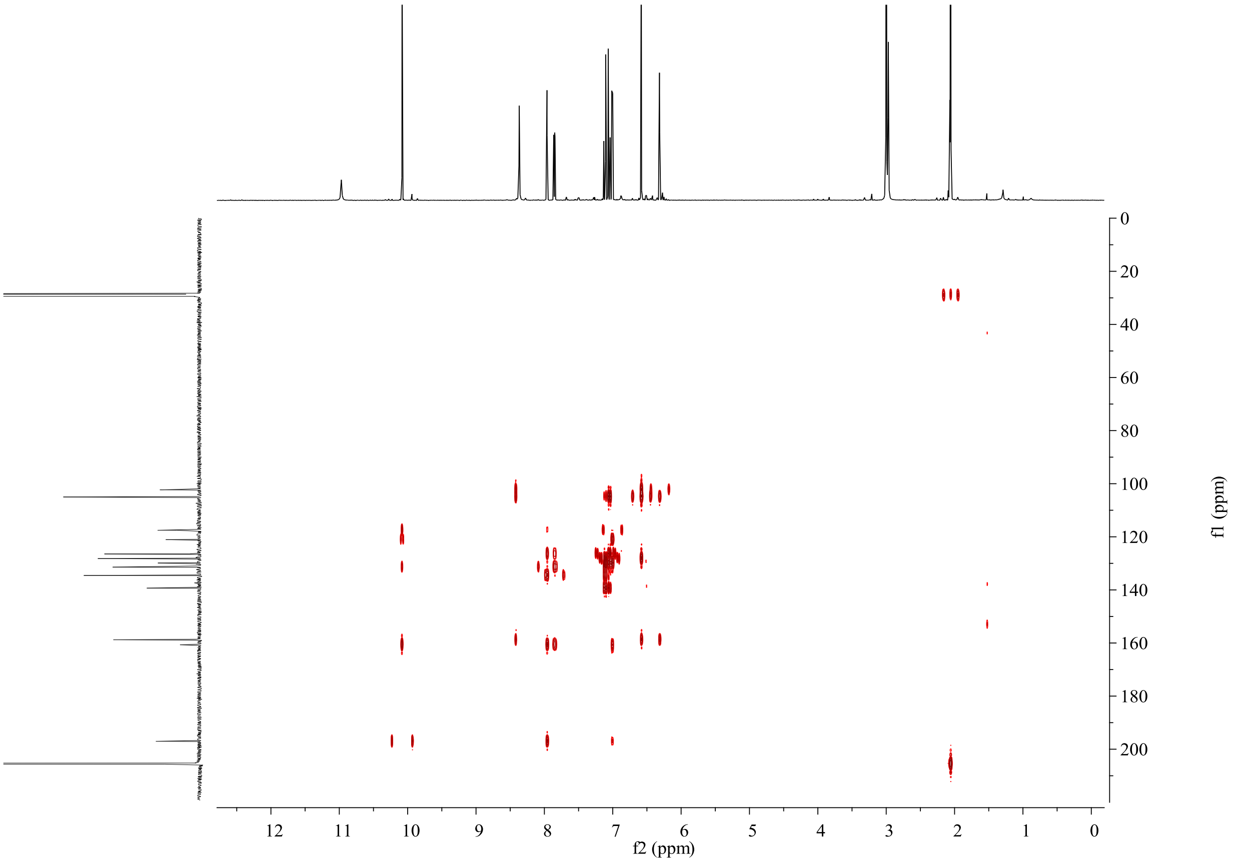

Figure S6. Expansion of (600/150 MHz) of compound **1** in $(CD_3)_2CO$.

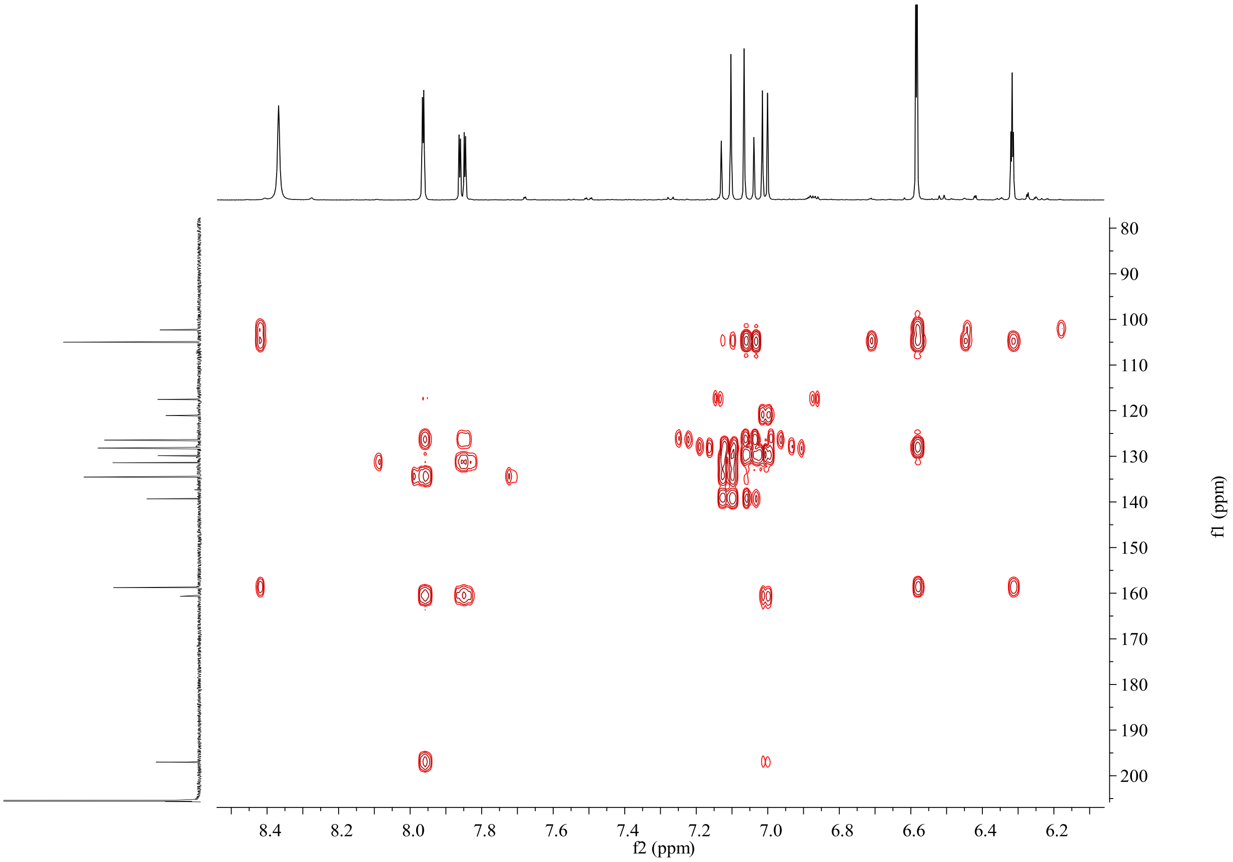

Figure S7. HRESIMS spectrum of compound **1**.

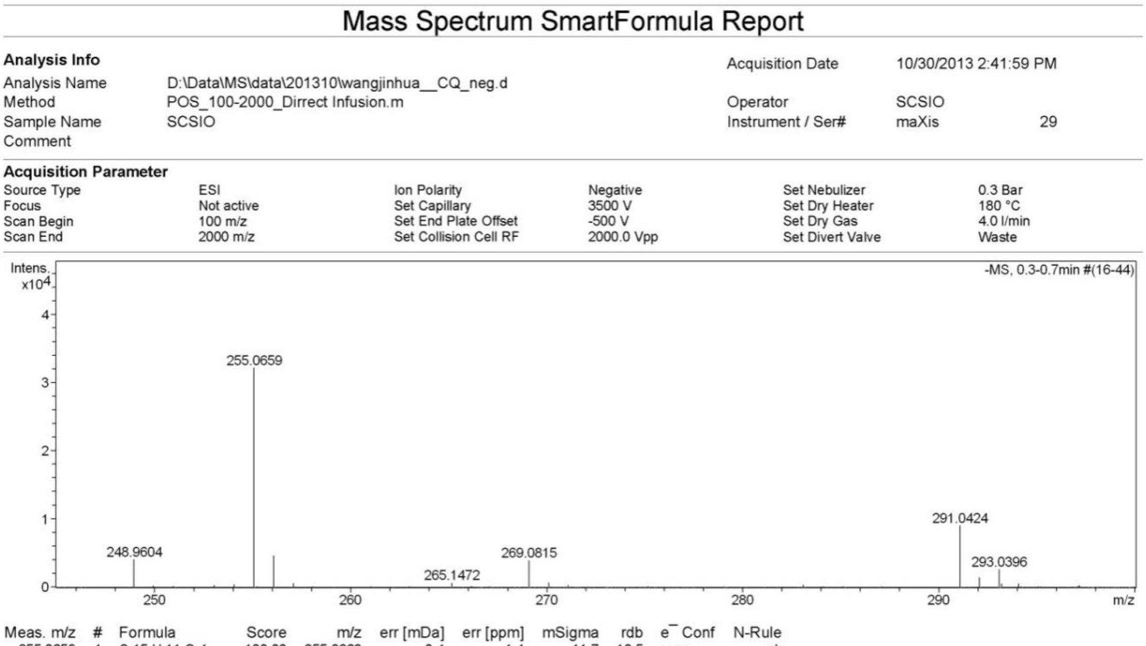

Meas. m/z	#	Formula	Score	m/z	err [mDa]	err [ppm]	mSigma	rdb	e⁻ Conf	N-Rule
255.0659	1	C 15 H 11 O 4	100.00	255.0663	0.4	1.4	11.7	10.5	even	ok
291.0424	1	C 15 H 12 Cl O 4	100.00	291.0430	0.6	2.1	22.0	9.5	even	ok

Figure S8. IR spectrum of compound **1**.

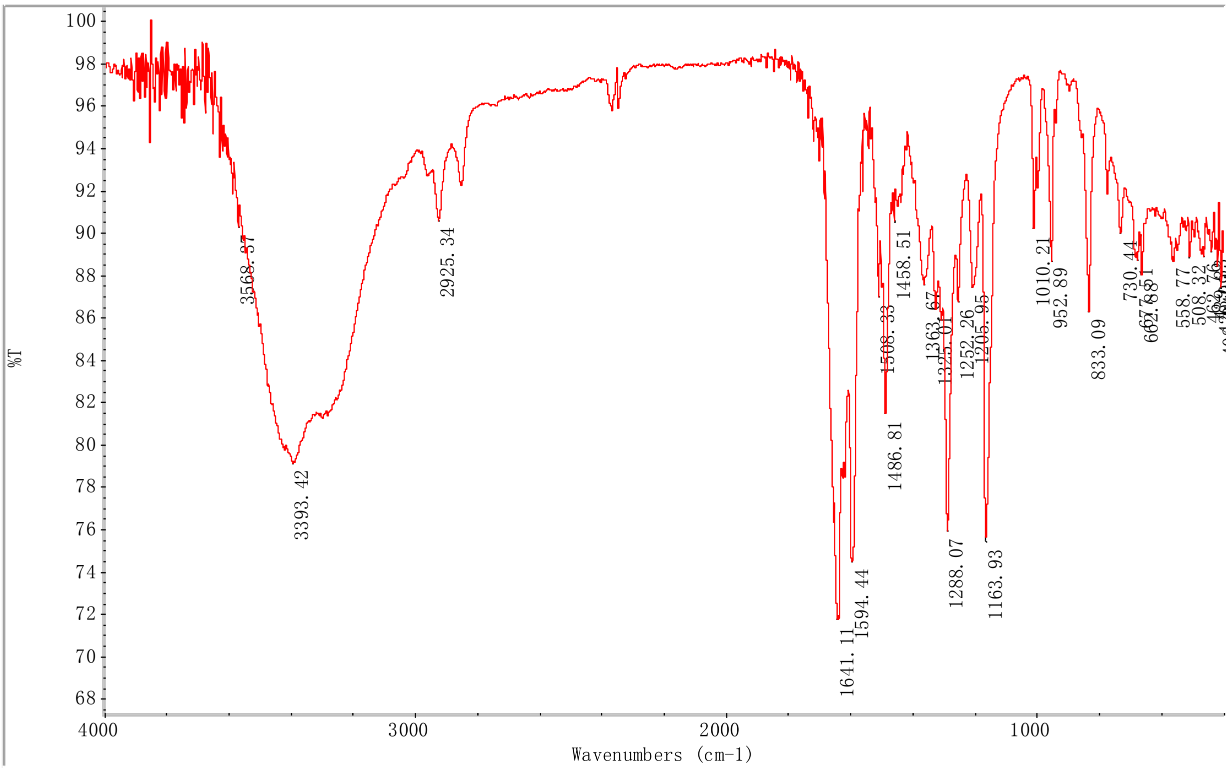

Figure S9. ^{1}H NMR spectrum (600 MHz) of compound **2** in $(CD_3)_2CO$.

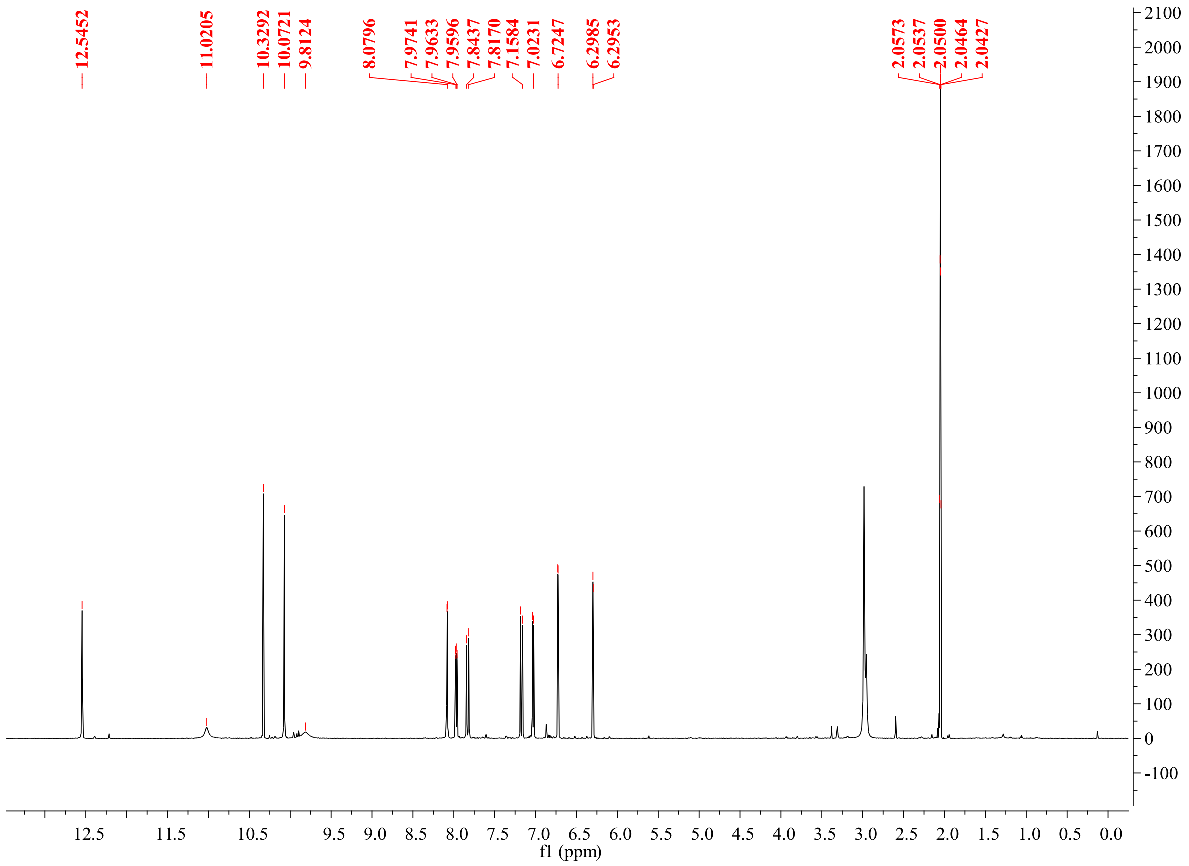

Figure S10. Expansion of ^{1}H NMR spectrum (600 MHz) of compound **2** in $(CD_3)_2CO$.

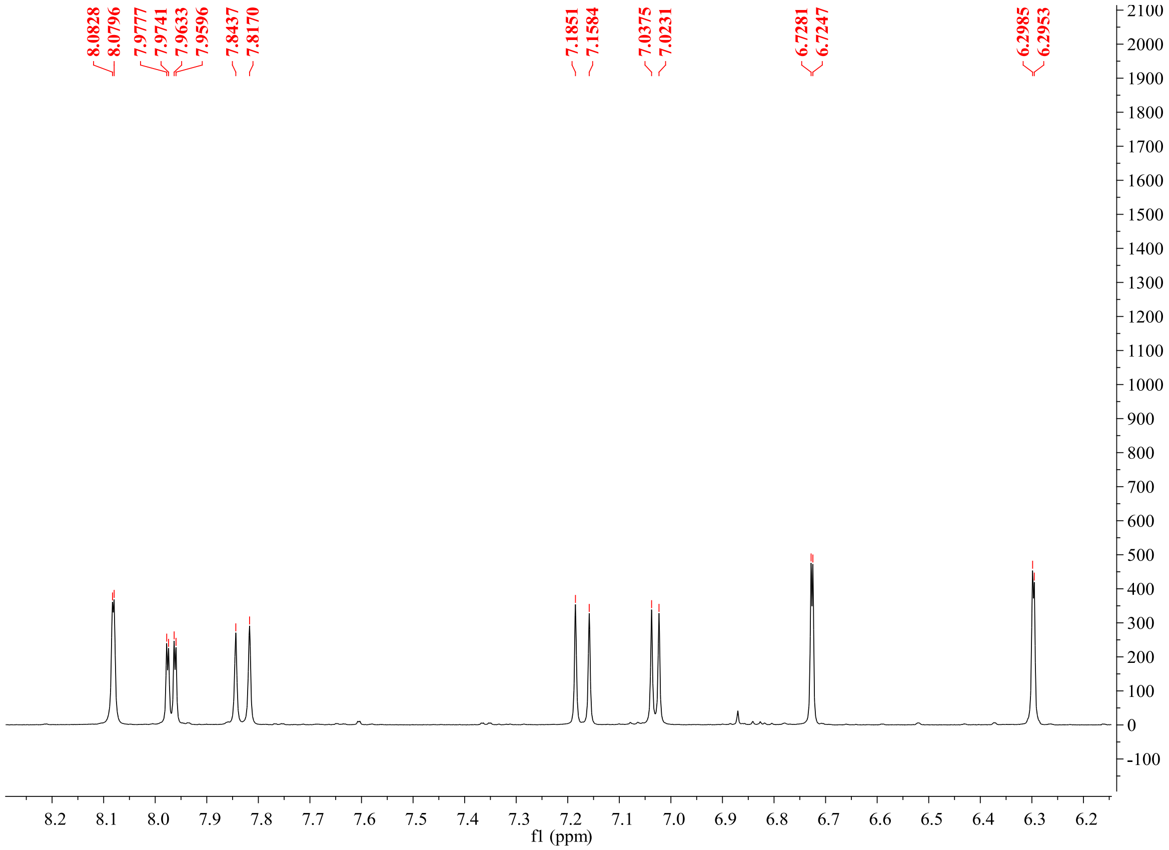

Figure S11. ^{13}C NMR spectrum (150 MHz) of compound **2** in $(CD_3)_2CO$.

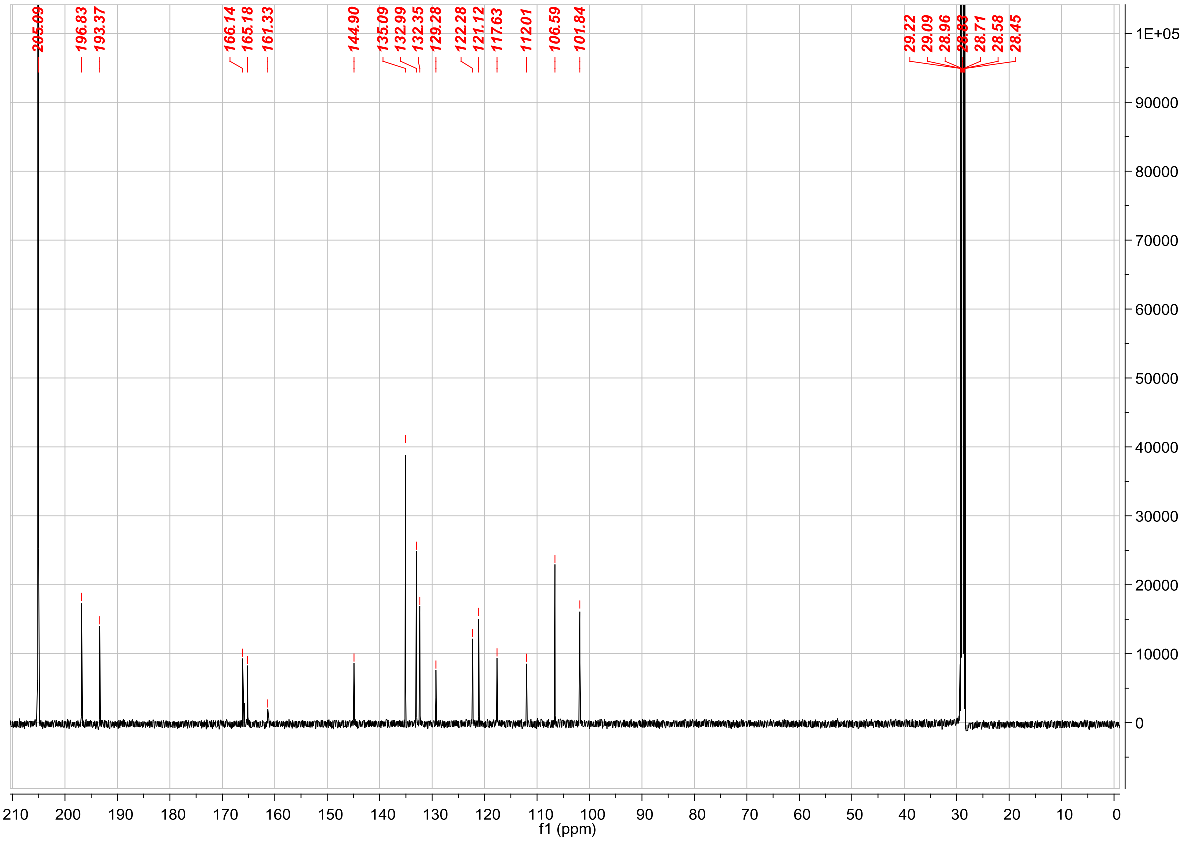

Figure S12. HSQC spectrum (600/150 MHz) of compound **2** in $(CD_3)_2CO$.

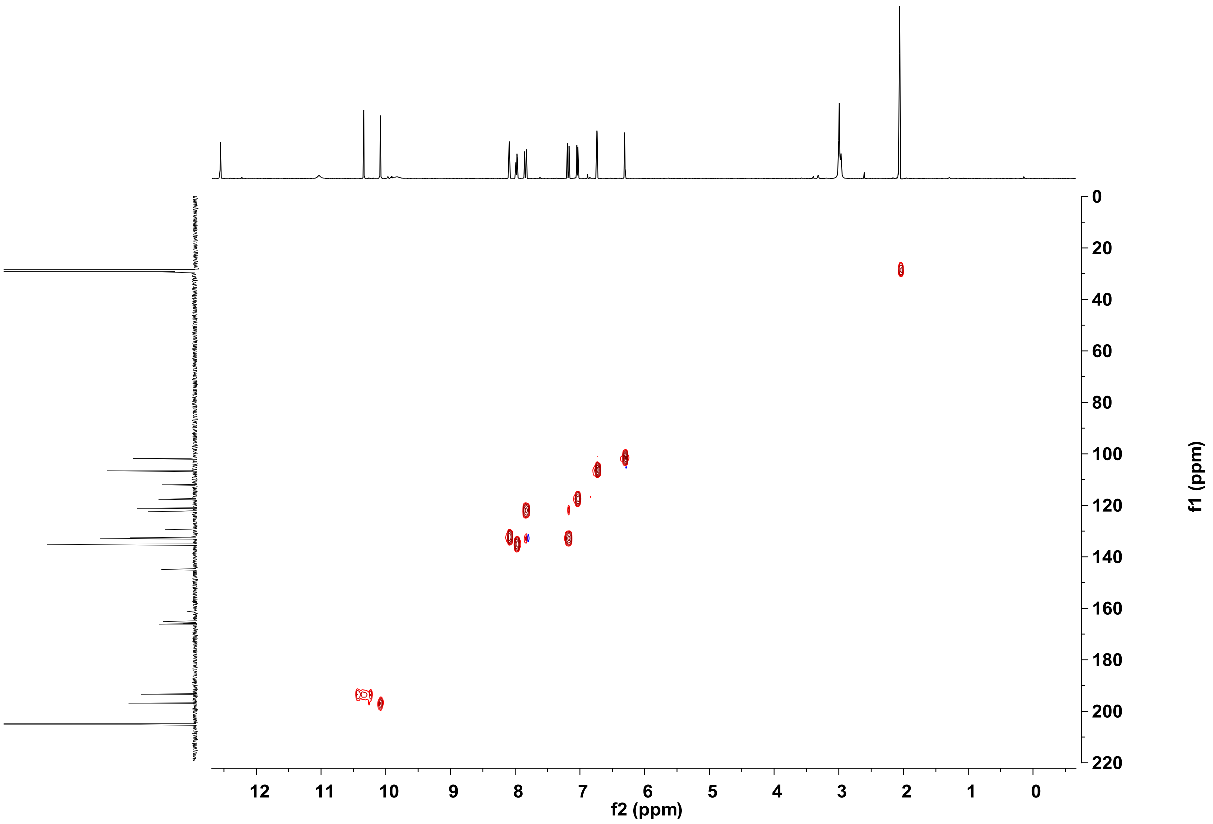

Figure S13. HMBC spectrum (600/150 MHz) of compound **2** in $(CD_3)_2CO$.

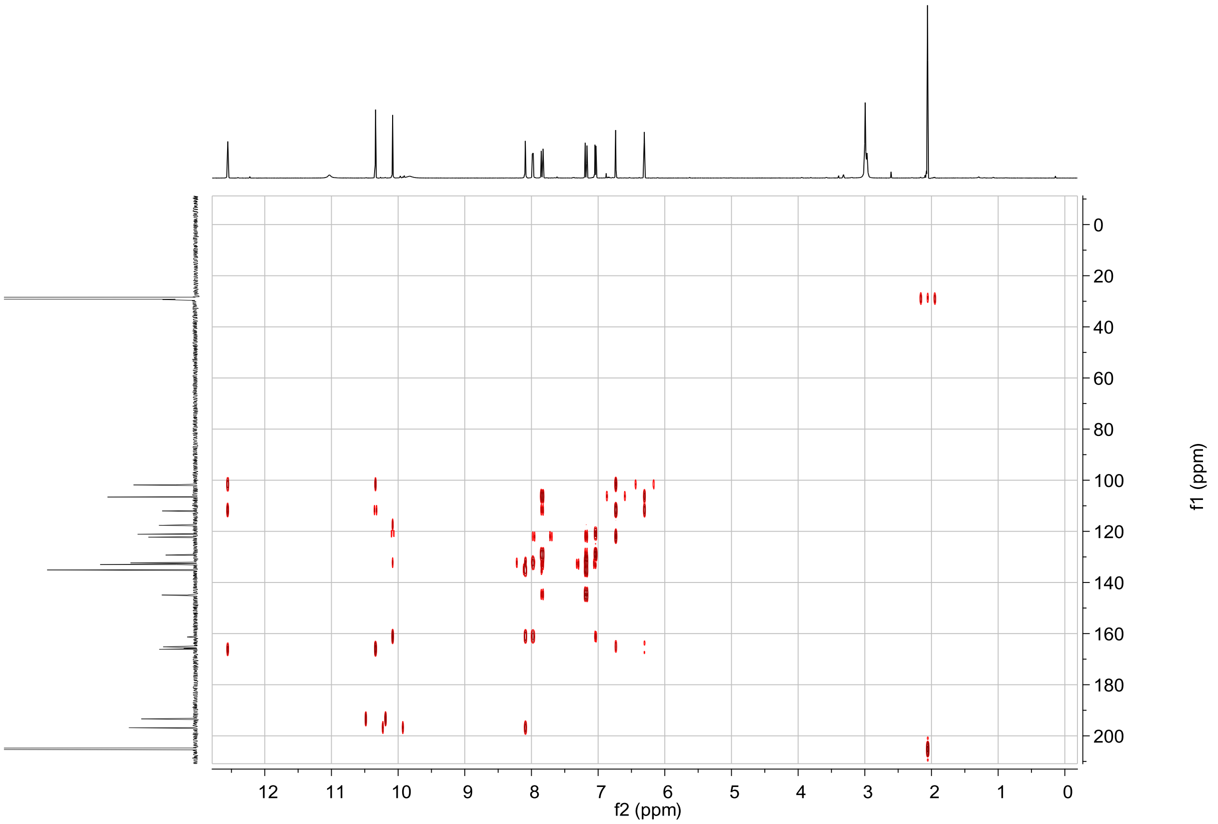

Figure S14. Expansion of HMBC spectrum(600/150 MHz) of compound **2** in $(CD_3)_2CO$.

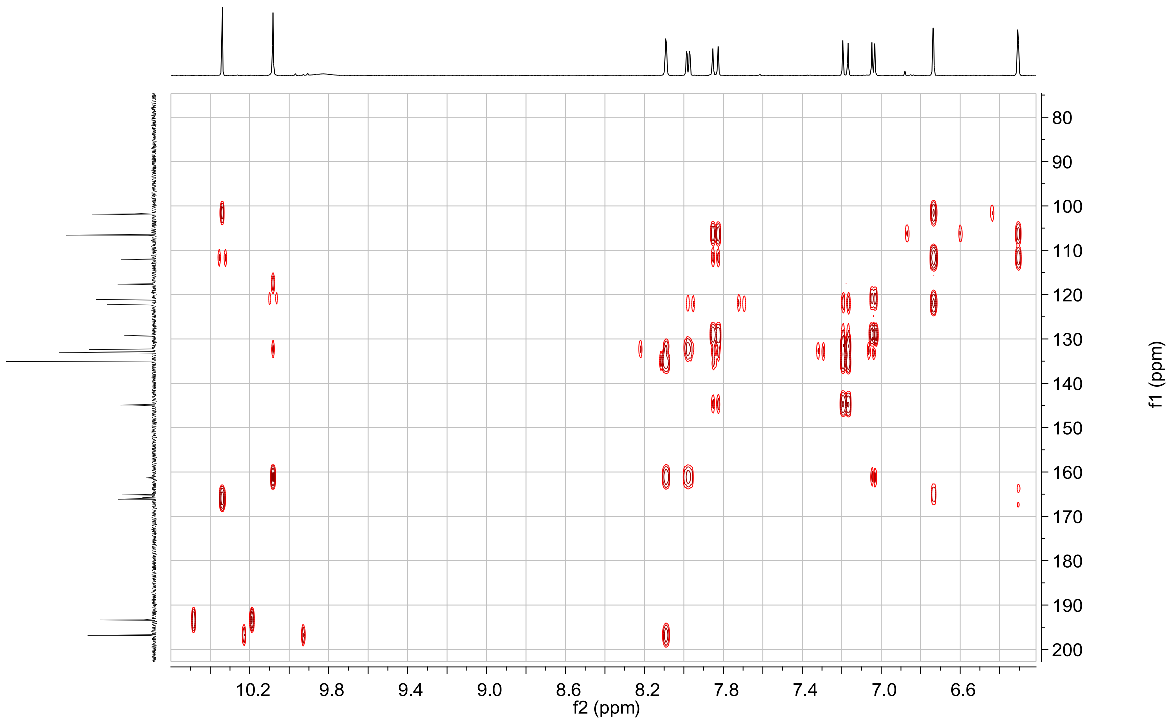

Figure S15. HRESIMS spectrum of compound **2**.

Mass Spectrum SmartFormula Report

Analysis Info

Analysis Name	D:\Data\MS\data\201312\wangjinhua-LZG_neg.d	Acquisition Date	12/17/2013 2:52:20 PM
Method	POS_100-2000_Dirrect Infusion.m	Operator	SCSIO
Sample Name	SCSIO	Instrument / Ser#	maXis 29
Comment			

Acquisition Parameter

Source Type	ESI	Ion Polarity	Negative	Set Nebulizer	0.3 Bar
Focus	Not active	Set Capillary	3500 V	Set Dry Heater	180 °C
Scan Begin	100 m/z	Set End Plate Offset	-500 V	Set Dry Gas	4.0 l/min
Scan End	2000 m/z	Set Collision Cell RF	2000.0 Vpp	Set Divert Valve	Waste

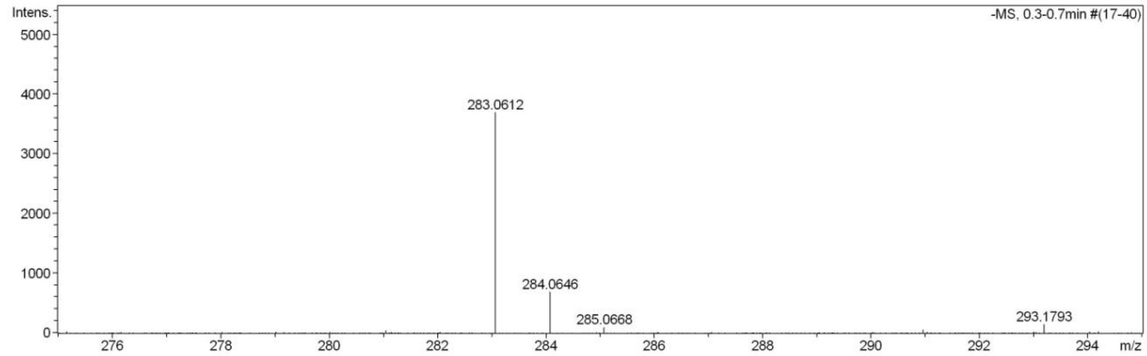

Meas. m/z	#	Formula	Score	m/z	err [mDa]	err [ppm]	mSigma	rdb	e⁻ Conf	N-Rule
283.0612	1	C 16 H 11 O 5	100.00	283.0612	-0.0	-0.0	7.2	11.5	even	ok
567.1289	1	C 32 H 23 O 10	91.42	567.1297	0.8	1.4	5.7	21.5	even	ok

Bruker Compass DataAnalysis 4.0 printed: 12/17/2013 2:58:42 PM Page 1 of 1

Figure S16. IR spectrum of compound **2**.

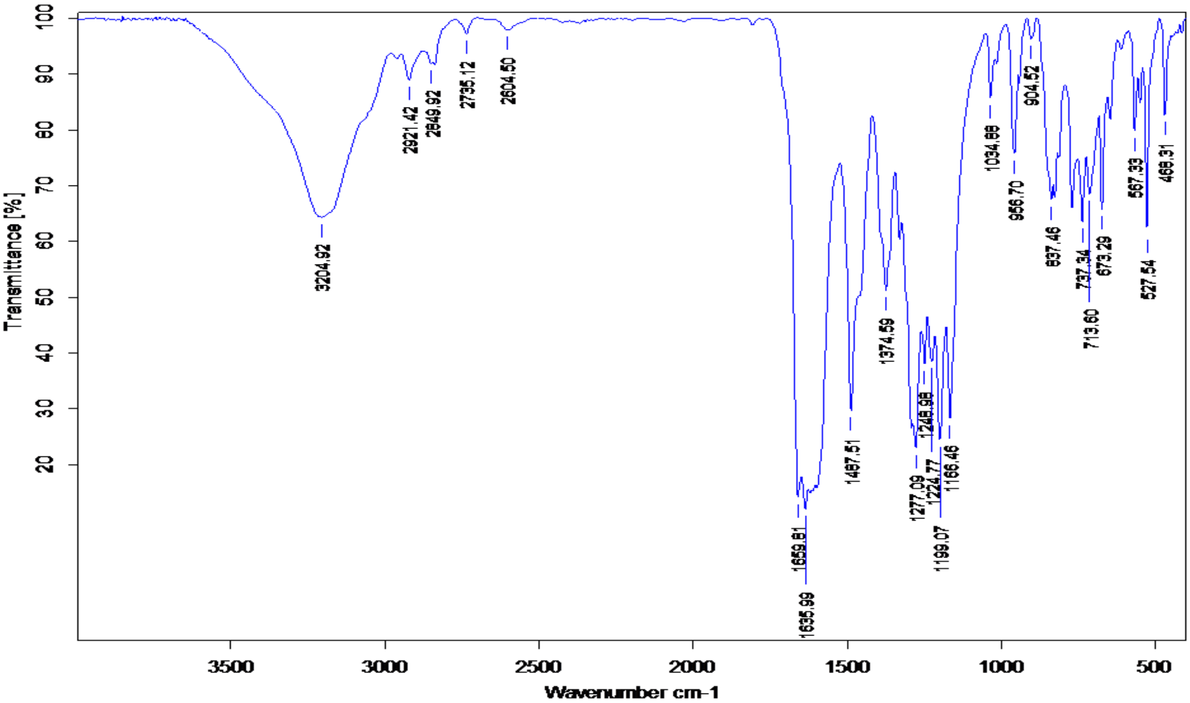

Figure S17. ^{1}H NMR spectrum (600 MHz) of compound **3** in $(CD_3)_2CO$.

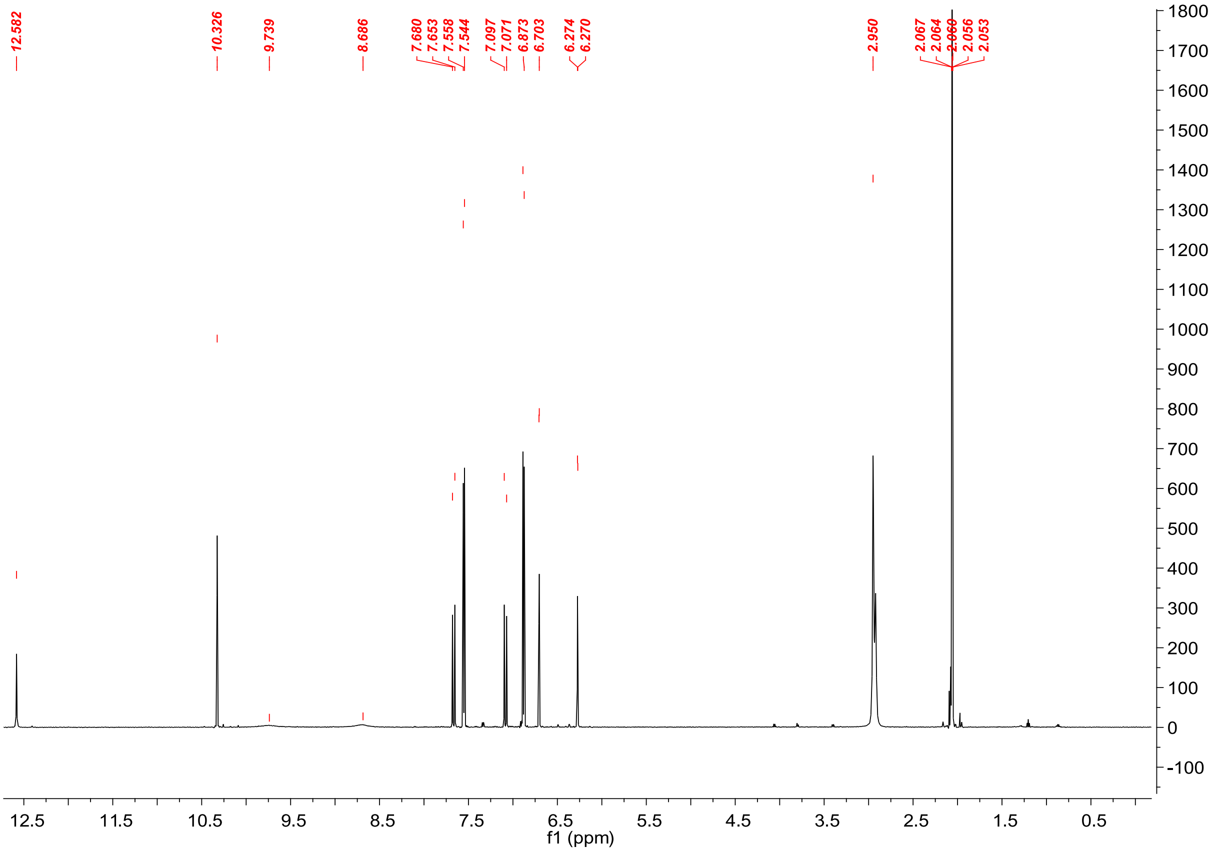

Figure S18. Expansion of ^{1}H NMR spectrum (600 MHz) of compound **3** in $(CD_3)_2CO$.

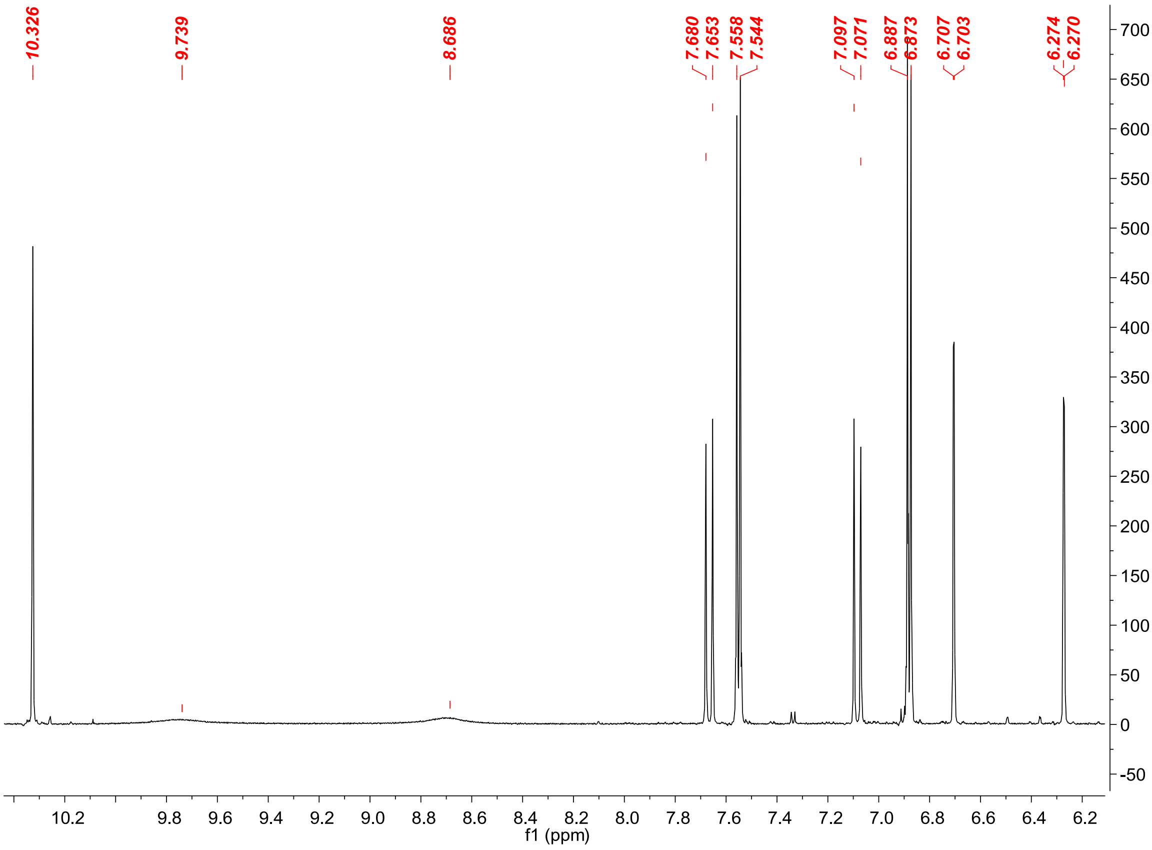

Figure S19. ^{13}C NMR spectrum (150 MHz) of compound **3** in $(CD_3)_2CO$.

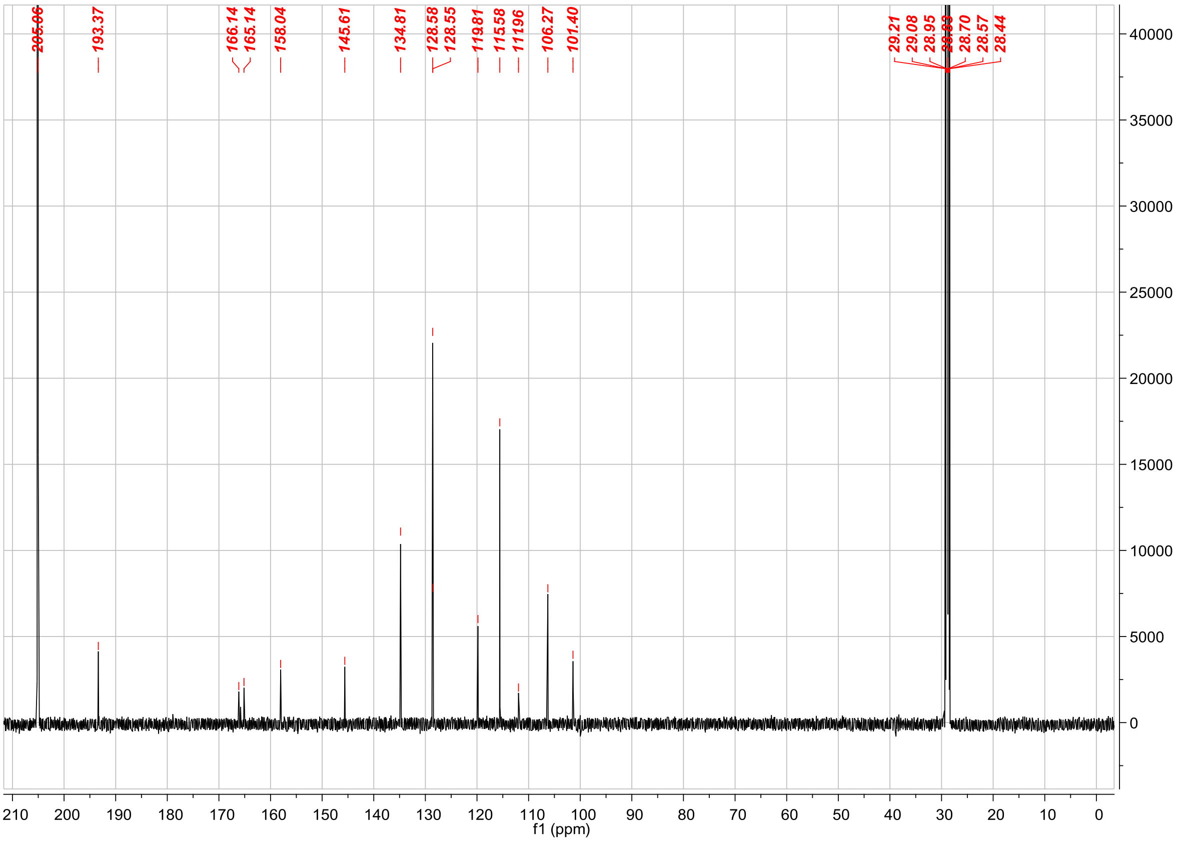

Figure S20. HSQC spectrum (600/150 MHz) of compound **3** in $(CD_3)_2CO$.

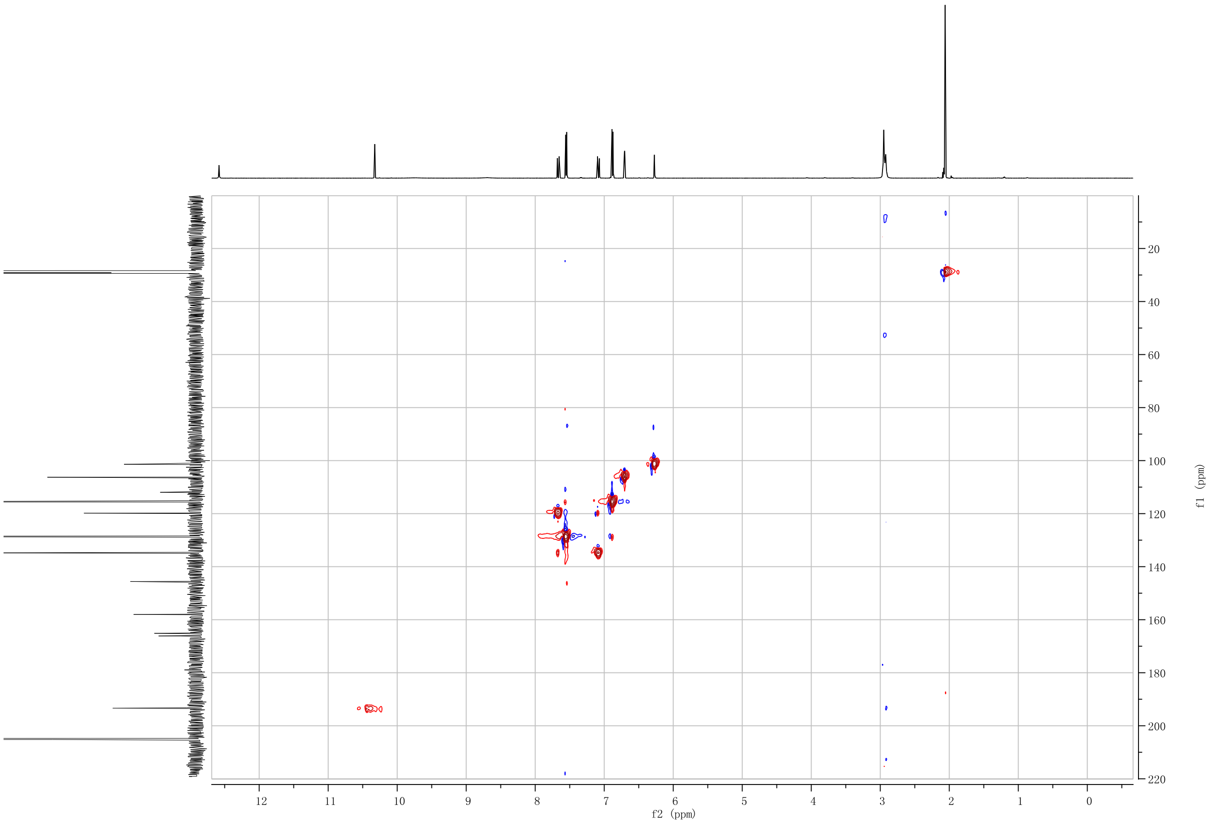

Figure S21. HMBC spectrum (600/150 MHz) of compound **3** in $(CD_3)_2CO$.

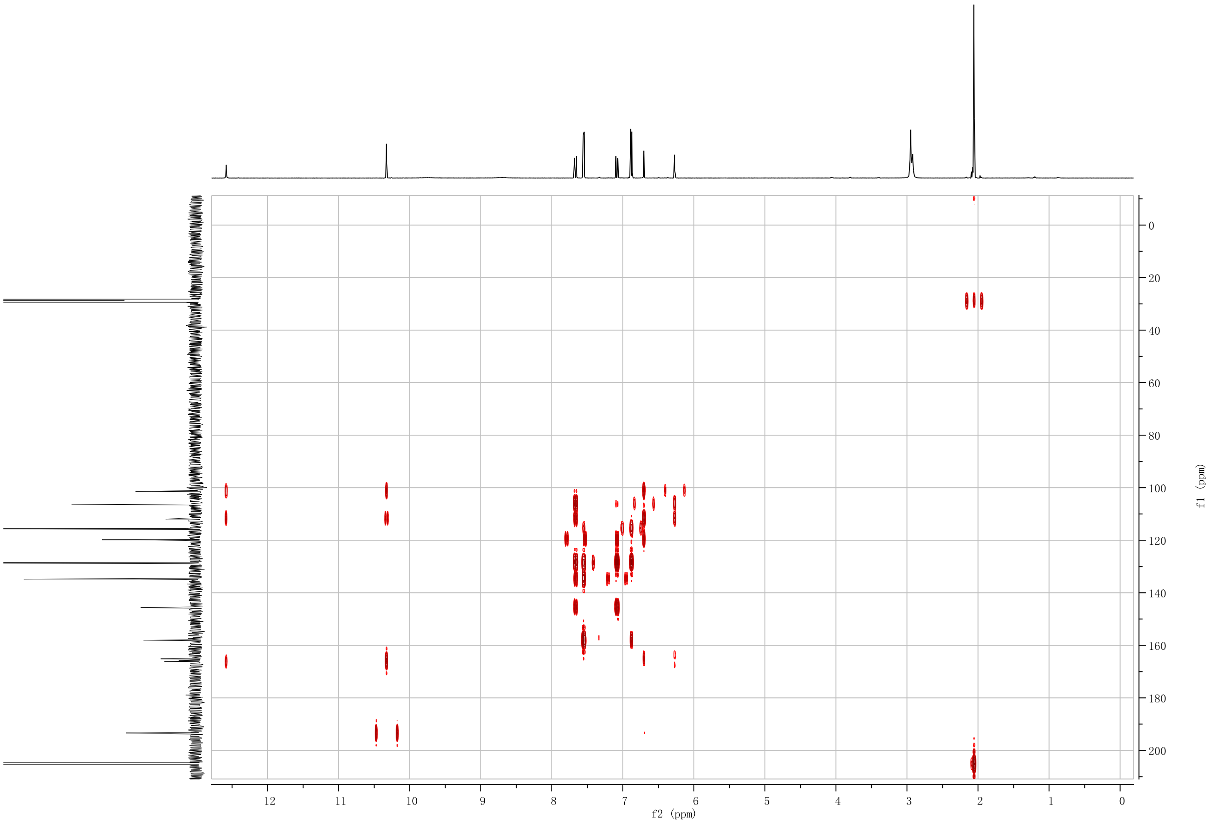

Figure S22. Expansion of HMBC spectrum(600/150 MHz) of compound **3** in $(CD_3)_2CO$.

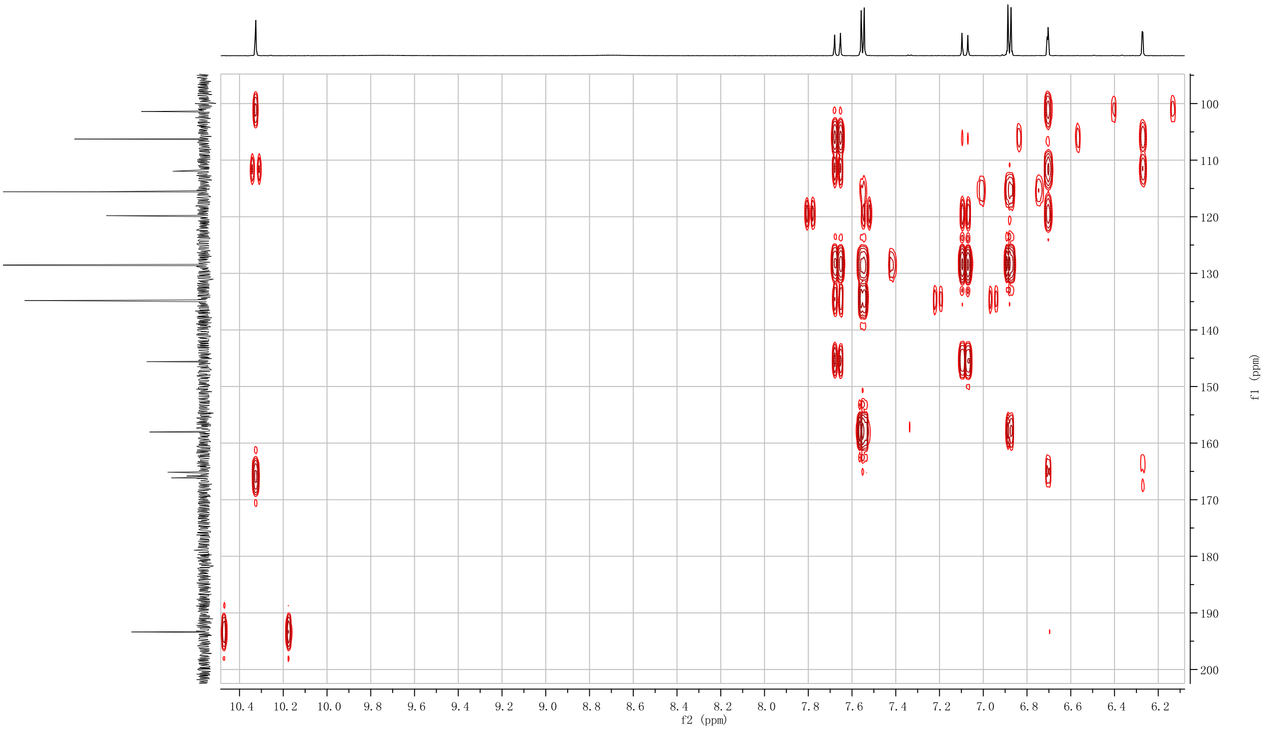

Figure S23. HRESIMS spectrum of compound **3**.

Mass Spectrum SmartFormula Report

Analysis Info

Analysis Name	D:\Data\MS\data\201310\wangjinhua__CP_neg.d	Acquisition Date	10/30/2013 2:38:47 PM
Method	POS_100-2000_Dirrect Infusion.m	Operator	SCSIO
Sample Name	SCSIO	Instrument / Ser#	maXis 29
Comment			

Acquisition Parameter

Source Type	ESI	Ion Polarity	Negative	Set Nebulizer	0.3 Bar
Focus	Not active	Set Capillary	3500 V	Set Dry Heater	180 °C
Scan Begin	100 m/z	Set End Plate Offset	-500 V	Set Dry Gas	4.0 l/min
Scan End	2000 m/z	Set Collision Cell RF	2000.0 Vpp	Set Divert Valve	Waste

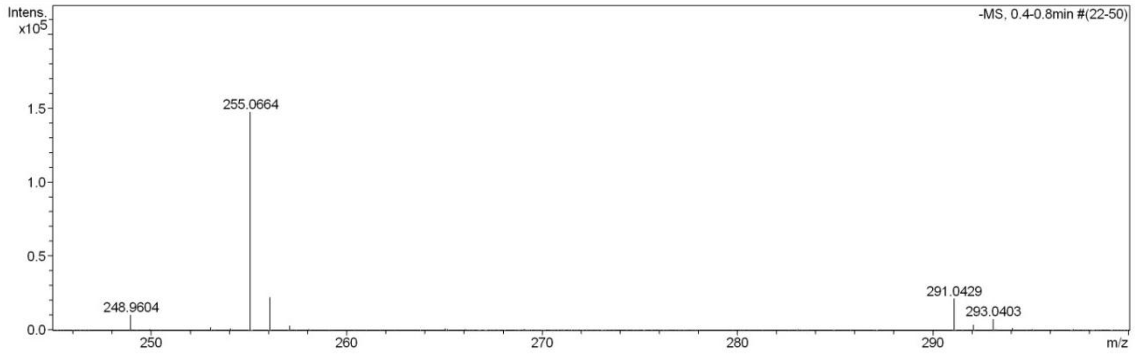

Meas. m/z	#	Formula	Score	m/z	err [mDa]	err [ppm]	mSigma	rdb	e⁻ Conf	N-Rule
255.0664	1	C 15 H 11 O 4	100.00	255.0663	-0.1	-0.5	7.4	10.5	even	ok
291.0429	1	C 15 H 12 Cl O 4	100.00	291.0430	0.0	0.1	4.3	9.5	even	ok
511.1396	1	C 30 H 23 O 8	100.00	511.1398	0.2	0.4	17.2	19.5	even	ok
547.1162	1	C 30 H 24 Cl O 8	100.00	547.1165	0.3	0.6	34.7	18.5	even	ok

Bruker Compass DataAnalysis 4.0 printed: 10/30/2013 2:51:43 PM Page 1 of 1

Figure S24. IR spectrum of compound **3**.

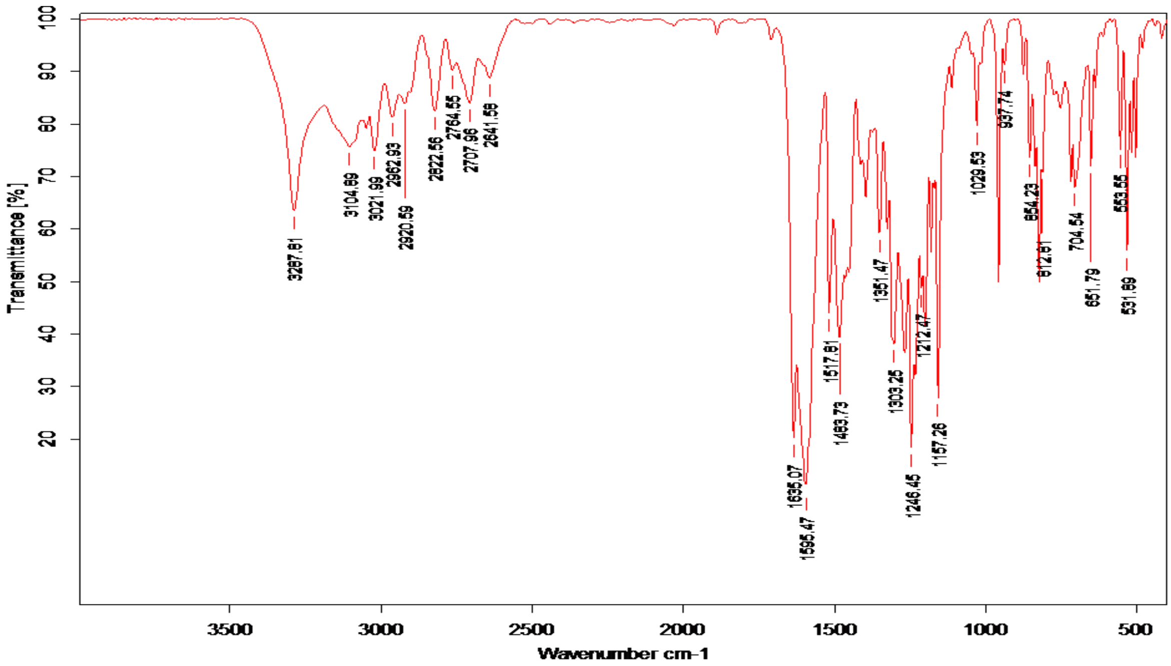

© 2014 by the authors; licensee MDPI, Basel, Switzerland. This article is an open access article distributed under the terms and conditions of the Creative Commons Attribution license (http://creativecommons.org/licenses/by/3.0/).
